# A comparison of two distinct murine macrophage gene expression profiles in response to *Leishmania amazonensis *infection

**DOI:** 10.1186/1471-2180-12-22

**Published:** 2012-02-09

**Authors:** Christian M Probst, Rodrigo A Silva, Juliana P B Menezes, Tais F Almeida, Ivana N Gomes, Andréia C Dallabona, Luiz S Ozaki, Gregory A Buck, Daniela P Pavoni, Marco A Krieger, Patrícia S T Veras

**Affiliations:** 1 Laboratório de Genômica Funcional, Instituto Carlos Chagas, ICC-FIOCRUZ, Paraná, Brazil; 2 Laboratório de Patologia e Biointervenção, CPqGM-FIOCRUZ, Bahia, Brazil; 3 Center for the Study of Biological Complexity, Virginia Commonwealth University, Richmond, VA, USA

## Abstract

**Background:**

The experimental murine model of leishmaniasis has been widely used to characterize the immune response against *Leishmania*. CBA mice develop severe lesions, while C57BL/6 present small chronic lesions under *L. amazonensis *infection. Employing a transcriptomic approach combined with biological network analysis, the gene expression profiles of C57BL/6 and CBA macrophages, before and after *L. amazonensis *infection in vitro, were compared. These strains were selected due to their different degrees of susceptibility to this parasite.

**Results:**

The genes expressed by C57BL/6 and CBA macrophages, before and after infection, differ greatly, both with respect to absolute number as well as cell function. Uninfected C57BL/6 macrophages express genes involved in the deactivation pathway of macrophages at lower levels, while genes related to the activation of the host immune inflammatory response, including apoptosis and phagocytosis, have elevated expression levels. Several genes that participate in the apoptosis process were also observed to be up-regulated in C57BL/6 macrophages infected with *L. amazonensis*, which is very likely related to the capacity of these cells to control parasite infection. By contrast, genes involved in lipid metabolism were found to be up-regulated in CBA macrophages in response to infection, which supports the notion that *L. amazonensis *probably modulates parasitophorous vacuoles in order to survive and multiply in host cells.

**Conclusion:**

The transcriptomic profiles of C57BL/6 macrophages, before and after infection, were shown to be involved in the macrophage pathway of activation, which may aid in the control of *L. amazonensis *infection, in contrast to the profiles of CBA cells.

## Background

Several factors related to the pathogen itself greatly influence the severity and clinical manifestation of infectious diseases, including parasite pathogenicity and virulence, as well as a variety of other factors related to the host's state of general health and genetic background [1-4]. Functional genomics is an important tool to study host-pathogen interactions, since it gives insight into the molecular mechanisms that control the onset of disease [5-7].

The cutaneous leishmaniasis murine model has been widely used to characterize the immune response against *Leishmania*. The association between resistance to *Leishmania major *and cell differentiation in CD4^+ ^Th1 lymphocytes has been well documented [8,9]. The immune response to *L. amazonensis *varies in accordance with the genetic background of the host. *L. amazonensis *causes severe lesions at cutaneous inoculation sites in the highly susceptible CBA and BALB/c mouse strains [4,10,11], while this same parasite causes chronic non-healing lesions in *L. major*-resistant strains, such as C57BL/6, C3H and C57BL/10 [10,12-14]. In response to infection by *L. amazonensis*, highly susceptible BALB/c mice mount a Th2-type of immune response, while C57BL/6 mice develop a non-Th1-type of immune response [15].

Macrophages are immune cells involved in the early events of pathogen infection [3,16]. *Leishmania *spp. parasites are delivered to the mammal dermis in the form of metacyclic promastigotes where they are phagocytosed [17]. Some *Leishmania *species, such as *L. amazonensis*, can survive and proliferate inside macrophages by modulating host cell killing mechanisms, regardless of microbicidal molecule production [3]. Following uptake, the surviving promastigotes differentiate into amastigotes and multiply within parasitophorous vacuoles [18].

Several studies have demonstrated that the survival of *Leishmania *spp. is associated with slight modifications in macrophage gene expression [6,19-21]. Over the last 10 years, several studies have presented evidence that *Leishmania *species do not adequately induce classical macrophage activation [19,20]. Moreover, a recent study found that these parasites down- and up-regulate similar numbers of proinflammatory response genes in human macrophages, as well as activate a gene that is compatible with an alternative phenotype [21]. Other authors have recently demonstrated that *L. amazonensis *is able to induce a transcriptional signature that resembles deactivation yet also appears similar to an alternative macrophage activation signature [22]. Interestingly, these authors showed that *L. amazonensis *directs macrophage response towards lipid and polyamine pathways by activating parasite- and host tissue-protective processes [22].

The role that host genetic factors play in the outcome of pathogen infection has also been studied using microarray analysis [23,24]. In addition, several studies have compared the gene expression profiles of cells [23,24] and tissues [25] from a variety of mouse strains in response to several pathogens. However, no studies have yet attempted to compare the transcriptional signatures of uninfected macrophages from two distinct murine genetic backgrounds, nor the transcriptional programs of a distinct macrophage lineage in response to a single *Leishmania *species.

The present study employed a transcriptomic approach combined with biological network analysis to highlight the differences between the responses of murine macrophages from two inbred mouse strains to *L. amazonensis *infection. C57BL/6 and CBA strains were selected due to their divergent degrees of susceptibility to this parasite [4,12]. The expression profiles of more than 12,000 murine genes were evaluated in each mouse strain before and after infection in vitro. The authors identified the genes that were differentially expressed between uninfected C57BL/6 and CBA macrophages, thereby establishing baseline levels of differential expression. We then attempted to investigate modulations in macrophage gene expression, before and after infection, within a given mouse strain. We showed that the transcriptional profile of uninfected C57BL/6 macrophages differed from that of CBA macrophages with respect to the modulation of genes involved in the macrophage pathway of activation. In response to infection, C57BL/6 macrophages up-regulate genes related to controlling infection, while CBA cells up-regulate genes involved in lipid metabolism. These findings provide evidence that C57BL/6 macrophages' transcriptional profiles may help in the control of *L. amazonensis *infection, in contrast to the profiles of CBA cells.

## Methods

### Mice

All experiments were performed according to the guidelines of the Institutional Review Board on Animal Experimentation at the Oswaldo Cruz Foundation - CPqGM/FIOCRUZ. Male and female CBA mice, 6-12 weeks old, were provided by the Animal Care Facility at CPqGM/FIOCRUZ. The animals were housed under specific pathogen-free conditions, fed commercial rations and given water ad libitum.

### Parasites

The *L. amazonensis *(strain MHOM/Br88/Ba-125) promastigotes used in this study were grown in axenic culture for up to seven passages, suspended in Schneider's complete medium (Gibco, Grand Island, NY, USA) supplemented with 10% inactivated fetal calf serum and 50 μg/mL of gentamicin (Sigma, St. Louis, MO, USA). All parasite cultures were washed three times in a saline solution, counted, adjusted and added to macrophage cultures at a ratio of 10:1.

### Macrophage cultures

Inflammatory peritoneal macrophages were elicited using a 3 mL intraperitoneal injection of 3% thioglycolate solution (Sigma) in C57BL/6 or CBA mice. After 96 h, all animals were euthanized and the elicited peritoneal macrophages were obtained as previously described [3]. The cells were suspended in complete Dulbecco's Modified Eagle's Medium (DMEM) (Gibco) [DMEM supplemented with 10% fetal bovine serum (Gibco), 2 g/L sodium bicarbonate (Sigma), 25 mM HEPES (Sigma), 1 mM glutamine (Sigma) and 0.2% ciprofloxacin (Halexistar, Goiania, GO, BR)] and distributed in 6-well plates at a concentration of 1 × 10^7 ^macrophages per well. Cultures were subsequently incubated overnight at 37°C in 5% CO_2_.

### Macrophage infection

The inflammatory peritoneal macrophage cultures were infected for 12 h with *L. amazonensis *stationary phase promastigotes. Cell cultures were then washed twice with saline to remove non-internalized parasites and reincubated for an additional six or 24 h before either RNA extraction or fixation with ethanol for 20 min followed by staining with hematoxylin and eosin (H&E). Each independent experiment was repeated three times for microarray analysis, and each experiment was performed at least three times in triplicate for microscopic analysis.

### Microarray analysis

Total RNA from uninfected or *L. amazonensis*-infected macrophages was prepared using Qiagen RNeasy mini-prep columns (Qiagen, Valencia, CA, USA) in accordance with manufacture protocols. The integrity of each RNA preparation was assessed using agarose gel electrophoresis. The RNA was reverse transcribed using Superscript II (Invitrogen, Carlsbad, CA, USA) in the presence of oligo(dT) primers linked to a T7 RNA polymerase promoter sequence (Proligo, La Jolla, CA, USA) to prime cDNA synthesis. After second-strand synthesis, biotinylated cRNA was produced by in vitro transcription using biotinylated UTP and CTP (Bioarray high-yield RNA transcript labeling kit, Enzo Diagnostics, Farmingdale, NY, USA) and purified with RNAeasy mini columns (Qiagen). The biotinylated cRNA was fragmented at 94°C for 30 min. For probe array hybridization and scanning, 16 μg of fragmented labeled cRNA was hybridized to the Murine Genome U74v2 GeneChip^® ^array (Affymetrix, Santa Clara, CA, USA), which contains nearly 400,000 probe sets covering approximately 12,000 different murine genes. Array scanning was performed using the Affymetrix^® ^GeneChip Scanner 3000 7 G and all images were analyzed using Microarray Analysis Software (Affymetrix v5.0). Experimental data are available online at ArrayExpress (E-MEXP-3448).

### Statistical analysis of differentially expressed genes among C57BL/6 and CBA macrophages

All microarray data were analyzed using the gcRMA library [26] from the Bioconductor project, using the R statistical software suite. Next, in order to identify differentially expressed genes, the SAM (Significance Analyses of Microarray) statistical package was used to compare the levels of gene expression among the following groups: (1) uninfected C57BL/6 and CBA macrophages; (2) *L. amazonensis*-infected C57BL/6 macrophages and uninfected cells; (3) *L. amazonensis*-infected CBA macrophages and uninfected cells; (4) *L. amazonensis*-infectedC57BL/6 and CBA macrophages. In order to enhance confidence in the statistical analysis of microarray data, experiment variables of incubation and infection time were not considered when comparing gene expression among groups (1) to (4). SAM software uses a modified *t*-test measurement which corrects for multiple comparisons by means of a False Discovery Rate (FDR) approach [27]. The q-values, or the minimum FDRs at which a statistical test may be called significant [28], have been provided for each differentially expressed gene in Tables S1, S2 and S3 (See Additional file 1: Table S1; Additional file 2: Table S2 and Additional file 3: Table S3, respectively). Finally, differentially expressed genes were analyzed and grouped in functional networks using the Ingenuity Pathway Analysis program v8.8 (IPA-Ingenuity Systems^®^, http://www.ingenuity.com). Possible networks and pathways were scored and modeled considering the sets of differentially expressed genes derived from the four comparisons described above. To calculate the probability of associations between genes from the functional networks and pathways generated by IPA^®^, Fisher's exact test was used with a 0.05 threshold value.

### Total macrophage mRNA extraction and mRNA quantification by RT-qPCR

In order to perform reverse transcriptase-quantitative polymerase chain reactions (RT-qPCR), RNA was initially extracted from uninfected or infected macrophages using a QIAGEN Mini Kit (RNAeasy) in accordance with manufacturer directions. An optical density reading was taken following extraction procedures and RNA integrity was verified using an agarose gel. Complementary DNA (cDNA) was synthesized by reverse transcription in a final volume of 20 μL containing 5 mM MgCl_2 _(Invitrogen), PCR buffer 1× (Invitrogen), deoxyribonucleotide triphosphates each at 1 mM (dNTPs - Invitrogen), 0.5 mM oligonucleotide (oligo d(T) - Invitrogen), 1 UI RNase inhibitor (RNase Out - Invitrogen), 2.5 UI reverse transcriptase (MuLVRT- Invitrogen) and 1 μg of sample RNA in RNAse-Free Distilled Water. All reaction conditions consisted of a single cycle at 42°C for 50 min, followed by 70°C for 15 min and, finally, 4°C for at least 5 min. Following reverse transcription, the synthesized cDNA was aliquoted and frozen at -20°C. The cDNA aliquots were later thawed and amplified by qPCR in order to perform gene quantification. All reactions were performed in a final volume of 20 μL containing SYBR green^® ^(Applied Biosystems, Foster City, CA, USA) commercial mix solution, composed of SYBR Green I Dye, AmpliTaq Gold^® ^DNA polymerase, dNTPs with dUTP, 10 ng cDNA, and 50 pmoles of reverse and forward primers for each evaluated gene (Invitrogen). qPCRs were run in a 7500 Real-Time PCR thermal cycler system (Applied Biosystems) and performed according to manufacturer's instructions, with variations occurring only with respect to melting temperature (Tm) for each pair of primers. Each sample was tested two or three times in duplicate. Table S4 (See Additional file 4: Table S4) lists the primer sequences used for each macrophage gene amplified by RT-qPCR, as well as Tm for each pair of primers.

### Analysis of mRNA quantification

Gene amplification results were obtained using Sequence Detection Software v1.3 (Applied Biosystems) with data expressed as mean values from experiments performed in duplicate. For each reaction, a serial dilution containing a mixture of cDNA from both uninfected and infected macrophages was used to generate a standard curve for gene expression quantification. Each gene's expression values were normalized against the respective value of the constitutive *gapdh1 *(glyceraldehyde 3-phosphate dehydrogenase) gene. The following comparisons of normalized gene expression were made: (1) C57BL/6 macrophages in relation to CBA macrophages; (2) *L. amazonensis*-infected C57BL/6 macrophages in relation to uninfected cells; (3) *L. amazonensis*-infected CBA macrophages in relation to uninfected cells. Resulting comparison values were expressed as mean values of log_2 _± SE from the two independent experiments in comparison (1), and three independent experiments in comparisons (2) and (3), all performed in duplicate. To determine the statistically significant differences in gene expression between all groups using RT-qPCR, the nonparametric Mann-Whitney test was used with a significance level of *p *≤ 0.05.

## Results and discussion

### Differences in transcription between uninfected C57BL/6 and CBA macrophages

**Figure 1:**
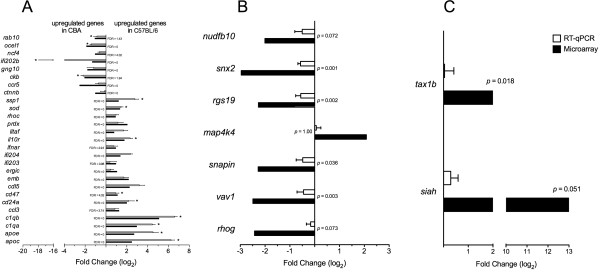
**Comparison of differentially expressed genes using microarray and RT-qPCR techniques**. RT-qPCR was used to verify the differential expression of randomly selected genes (n = 27) by uninfected C57BL/6 and CBA macrophages (A), by *L. amazonensis*-infected C57BL/6 macrophages in comparison to uninfected cells (n = 7) (B), and by *L. amazonensis*-infected CBA macrophages in comparison to uninfected cells (n = 2) (C). Figure 1 (A-C) depicts only genes that were successfully verified using RT-qPCR. Resulting comparison values are expressed as mean values of log_2 _± SE from two independent experiments in comparison (A), and three independent experiments in comparisons (B) and (C), all performed in duplicate. The nonparametric Mann-Whitney test was used for comparison between uninfected cells, and Stouffer method [29] was used to integrate the results from independent microarray and RT-qPCR analyses to determine significant differences between infected and uninfected cells (level of significance, *p *≤ 0.05)

In order to evaluate the influence of genetic factors on the outcome of *Leishmania *infection, the gene expression profiles from uninfected C57BL/6 and CBA macrophages were identified using an Affymetrix^® ^DNAmicroarray. Firstly, among the 12,000 genes analyzed using the Murine Genome U74v2 Genechip^®^, a total of 208 probe sets (See Additional file 1: Table S1) were found to be differentially expressed between the uninfected C57BL/6 and CBA macrophages with a 1.5 fold-change threshold and an estimated 5% FDR. All differential expression values are comparatively expressed as follows: a positive/negative value indicates that a given C57BL/6 macrophage exhibited a higher/lower level of expression than its CBA counterpart. Of these probe sets, 148 had higher expression levels in C57BL/6 macrophages (expressed as positive values) and 60 were found to be more highly expressed in CBA uninfected cells (expressed as negative values). In order to confirm these findings, a total of 27 genes were randomly selected and RT-qPCR was used to verify the differences in expression observed in the microarray analysis. Differential expression was confirmed in each of the 27 genes selected, and, among these, 13 genes showed statistically significant differences (Figure 1A).

### Increased levels of gene expression in uninfected C57BL/6 macrophages associated with cell death and lipid metabolism

**Figure 2:**
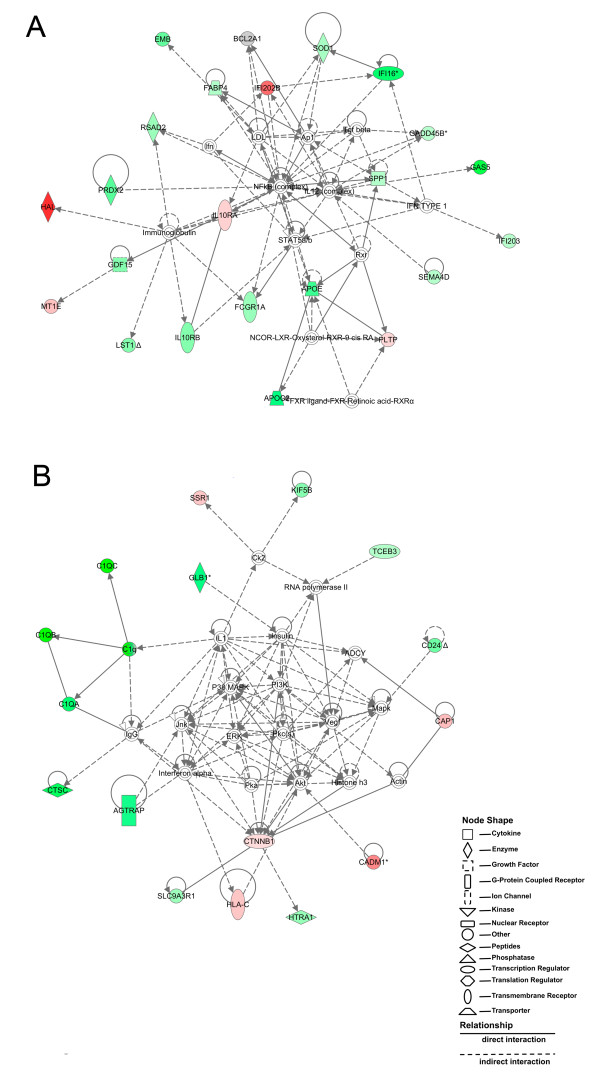
**Networks built using differentially expressed genes in uninfected macrophages from C57BL/6 and CBA mice**. C57BL/6 and CBA macrophages were cultured separately and then processed for microarray analysis as described in Materials and Methods. The cell death and lipid metabolism network (A) and the cell-cell signaling and interaction network (B) were modeled using Ingenuity Pathway Analysis software v8.8 (IPA-Ingenuity Systems^®^). The above networks are displayed as a series of nodes (genes or gene products) and edges (or lines, corresponding to biological relationships between nodes). Nodes are displayed using shapes that represent the functional class of the gene product as indicated in the key. Nodes marked in green were found to be highly expressed in C57BL/6 macrophages in comparison to CBA. Nodes marked in red were found to be highly expressed in CBA macrophages compared to C57BL/6. The unmarked nodes were not identified in our samples; however, IPA^® ^added them to the networks due to their high probability of involvement in a given network. The node color intensity is an indication of the degree of up-(green) or down-(red) regulation of genes observed in the biological network analysis from uninfected C57BL/6 macrophages compared to CBA cells. Solid lines denote direct interactions, whereas dotted lines represent indirect interactions between the genes represented in this network.

Using IPA-Ingenuity Systems^® ^v8.8 biological data analysis software, several functional networks and metabolic pathways were modeled from the differentially expressed genes by uninfected C57BL/6 and CBA macrophages. The cell death and lipid metabolism network had the highest probability of interrelated genes being differentially expressed (score 51). In this network, 17 out of the 22 genes identified by microarray analysis had higher levels of expression in C57BL/6 macrophages in comparison to CBA macrophages (Figure 2A). Among these, some encode proteins involved in lipid metabolism: *apoe *(+2.69) and *apoc2 *(+2.47). Both apolipoprotein E (Apoe) and apolipoprotein C (Apoc) are lipoproteins, mainly components of lipoprotein complexes, which are associated with proteins in plasma and the central nervous system [30].

Apoe regulates the metabolism of lipids by directing their transport, delivery, and distribution from one type of tissue or cell to another [30,31]. Alternatively, Apoe is also known to participate in the immune inflammatory response by scavenging reactive oxygen species (ROS). Accordingly, some genes that encode enzymes involved in antioxidant activity, such as *sod1 *(+1.34) and *prdx2 *(+2.05) were also expressed at higher levels in C57BL/6 macrophages. A previous study showed that peroxiredoxins (Prdxs) constitute a family of multifunctional antioxidant thiol-dependent peroxidases, which may modulate macrophage defense mechanisms against oxidative stress during inflammatory or infection events [32]. In this study, Bast et al. (2010) found higher levels of expression of peroxiredoxin mRNA and Prdx2 by C57BL/6 macrophages in response to stimulation with lipopolysaccharide (LPS) and IFN-γ, compared to BALB/c macrophages, which are known to be as susceptible as CBA macrophages to *L. amazonensis*. The proteins encoded by *prdx2 *and *apoe *may alternately play a role in apoptosis [33], in addition to *ifi204 *(+1.38), also known as *ifi16*, which encodes a transcriptional regulator, and *gdf15 *(+1.51), which encodes growth differentiation factor-15. It is possible that, with respect to uninfected CBA macrophages, the lower baseline levels of differential expression found among genes involved in apoptosis may affect the ability of these cells to control *L. amazonensis *infection [3].

Besides being a component of both high and very low-density lipoproteins, Apoc is known to readily accumulate in amyloid fibrils, inducing macrophage inflammatory responses, such as ROS production and TNF-α expression [34]. It is possible that the lower *apoc2 *expression levels found in uninfected CBA macrophages herein might be related to the low levels of TNF-α expression in IFN-γ-stimulated CBA macrophages in response to *L. amazonensis *infection demonstrated by a previous study [3].

Genes such as *chi3l3*/*chi3l4, fizz1*/*relm-α *and *arg1 *are considered to be signature markers of alternative macrophage activation in response to IL-4 stimulation [6]. Among these types of genes, *chi3l3*/*chi3l4 *(+3.028) was found to have increased differential expression in C57BL/6 macrophages. In addition, *il10ra *(-1.39), which encodes the ligand-binding subunit of the immune receptor for the IL-10 cytokine, is known to be involved in macrophage deactivation, and was found to have a lower level of expression in C57BL/6 macrophages. Accordingly, *fcgr1a *(+1.27), which encodes the high-affinity Fc-gamma receptor, participates in the innate immune response by promoting the clearance of pathogens and necrotic cells, and also was found to be more highly expressed in C57BL/6 macrophages.

By contrast, very few genes were identified as highly expressed in CBA macrophages compared to C57BL/6 (represented by negative expression values) in the cell death and lipid metabolism network (Figure 2A), such as *mt1 *(-0.99), which can have a protective effect on cells against apoptosis and oxidative stress responses; *hal *(-5.65), which participates in histidine catabolism; and *pltp *(-1.19), which is involved in lipid transport and metabolism.

### Increased levels of gene expression in uninfected C57BL/6 macrophages associated with the cell-cell signaling and interaction network

IPA^® ^identified several genes as part of the cell-cell signaling and interaction network (score 30) (Figure 2B): *c1qa *(+2.95), *c1qb *(+5.08) and *c1qc *(+5.04). These genes encode components of the complement cascade and all had higher expression levels in C57BL/6 macrophages. The classical pathway activation of complement elements constitutes events that are initiated by the binding of immune complexes to the C1 subcomponent, followed by subsequent C1q activation by serine proteases [35]. Constitutive synthesis of C1q in resident peritoneal macrophages suggests that *C1q *expression may be linked to the differentiation process in which blood monocytes become tissue macrophages [36]. Additionally, microorganism opsonization by C1q facilitates the phagocytosis of foreign particles during the innate immune response [37]. The production of anti-inflammatory mediators during proinflammatory responses is inhibited by C1q opsonization, which is followed by the phagocytosis of apoptotic cells [38].

In sum, the authors found significant differences in the baseline gene expression profiles of C57BL/6 macrophages compared to those of CBA cells, which suggests that the higher capacity of C57BL/6 macrophages to control *L. amazonensis *infection is related to the baseline transcriptional signature of these cells. These macrophages have genes involved in the deactivation pathway of macrophages which are expressed at lower levels, as well as higher expression levels of genes that encode proteins that play a role in the host immune inflammatory response, including several molecules involved in apoptosis in addition to phagocytic receptors that recognize pathogens and apoptotic cells.

#### Similarities between the expression profiles of genes related to apoptosis and stress response

Different genes with similar functions that are involved in specific cellular processes, e.g. apoptosis, immune and stress responses, were described as modulated by C57BL/6 and CBA macrophages. For instance, IFN-α/β-induced *ifi202 *gene expression was described by other authors as being induced in macrophages from several mouse strains, except C57BL/6 macrophages [39]. *ifi202 *participates in the immune response and composes the cell death and lipid metabolism network in the present study, this gene was shown to have a differential expression of -1.31 to -3.69 in C57BL/6 compared to CBA macrophages. This result was confirmed using RT-qPCR, which did not detect *ifi202 *expression in C57BL/6 macrophages. Additionally, other members of the *ifi200 *family, *ifi203 *(+0.96) and *ifi204 *(+1.38) genes were more highly expressed in C57BL/6 than in CBA cells. Taken together, these findings may suggest that different genes are responsible for triggering similar cellular processes, despite the distinct transcriptional signatures inherent in C57BL/6 and CBA macrophages.

### L. amazonensis infection triggers differentially expressed genes in macrophages from different genetic backgrounds

Macrophages' capacity to control parasite infection varies [3]. CBA macrophages are more susceptible to *L. amazonensis *infection than C57BL/6 macrophages. As depicted in Additional file 5: Figure S1, the percentage of infected CBA macrophages (78.50 ± 0.81% n = 3) was found to be 30% higher than in C57BL/6 macrophages (55.44 ± 3.86% n = 3) at 24 h after infection (*p *< 0.05, Mann Whitney test) (See Additional file 5: Figure S1A). In addition, the number of parasites per infected cell was also higher in CBA macrophages (3.42 ± 0.14 parasites/cell, n = 3) than in C57BL/6 (2.00 ± 0.06 parasites/cell, n = 3, *p *< 0.05, Mann-Whitney test) (See Additional file 5: Figure S1B). In order to analyze the response of macrophages to *L. amazonensis *infection, DNA microarray technology was used to compare differences in gene expression in response to parasite infection between infected and uninfected C57BL/6 or CBA macrophages. Firstly, the differential expression between infected and uninfected C57BL/6 or CBA macrophages was identified and tabulated (See Additional file 2: Table S2 and Additional file 3: Table S3). In response to *L. amazonensis *infection, C57BL/6 macrophages were observed to modulate 105 genes, while CBA macrophages modulated less than eleven times as many genes (n = 9). Next, to confirm these analyses, 12 out of the 105 differentially expressed genes in C57BL/6 macrophages were randomly selected for RT-qPCR verification. Differential expression was validated in seven of the 12 genes evaluated in these *L. amazonensis-*infected cells (Figure 1B). Conversely, only two of the six randomly selected genes that were differentially expressed by infected CBA cells were confirmed using RT-qPCR (Figure 1C).

In contrast to the relatively small number of differentially expressed genes detected in the present study, Osorio y Fortéa et al. (2009) encountered a considerable number of probe sets (1,248) with statistically significant differences in gene expression by *L. amazonensis*-infected BALB/c macrophages when compared to uninfected cells. Additionally, these authors found comparable fold-change values between the cDNA Affymetrix microarray analysis and the RTqPCR technique used for validation. There are several factors which may explain the differences in findings between these two studies: a) the present analysis collected peritoneal inflammatory macrophages from C57BL/6 and CBA mice, while Osorio y Fortéa et al. (2009) used BMMϕ from BALB/c mice; b) stationary-phase promastigotes were used to infect peritoneal macrophages in the present study, while Osorio y Fortéa et al. (2009) infected BMMϕ with amastigote forms of this same parasite; c) different versions of the Affymetrix gene chip were used in each study.

However, Zhang S. et al. (2010) showed that infection of BMMϕ with *L. mexicana*, a parasite species closely related to *L. amazonensis*, resulted in minimal changes in gene expression, which corroborates the findings of the present study. Furthermore, other reports have consistently described the global transcriptome of macrophages in response to *Leishmania *spp. infection in a similar fashion [6,19,20,40].

#### Genes involved in the host inflammatory response and apoptosis are modulated in C57BL/6 macrophages in response to *L. amazonensis* infection

**Figure 3:**
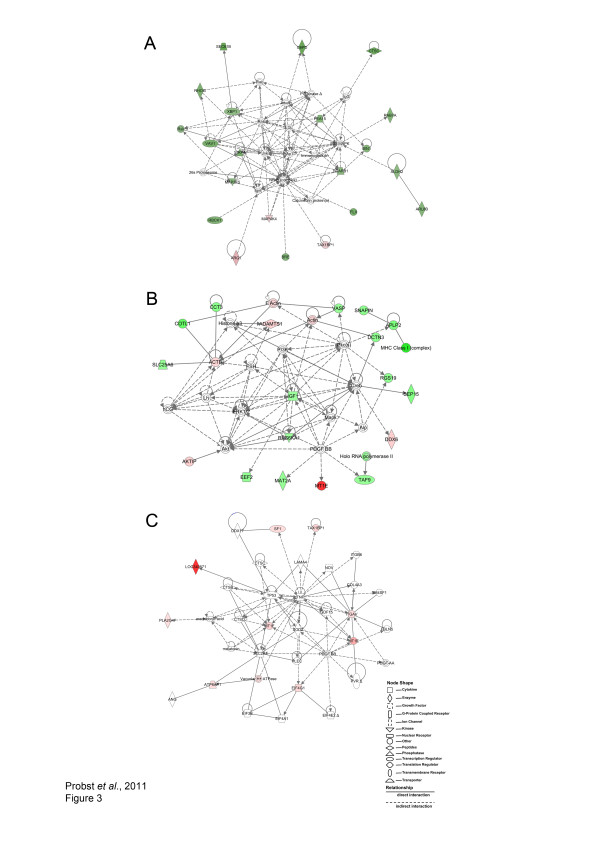
**Networks built using differentially expressed genes in *L. amazonensis-*infected and uninfected macrophages**. C57BL/6 or CBA macrophages were cultured, infected and processed for microarray analysis as described in Materials and Methods. Considering the modulated genes in C57BL/6 infected macrophages, the immunological disease and cell morphology network (A), as well as the protein synthesis, cellular development and cell death network (B) were modeled by IPA^®^. Considering the modulated genes in CBA infected macrophages, the lipid metabolism, cellular movement, and small molecule biochemistry network was built by IPA^® ^(C). C57BL/6 and CBA macrophages were cultured separately, then infected and processed for microarray analysis as described in Materials and Methods. Similar to Figure 2, the above networks are displayed as a series of nodes (genes or gene products) and edges (or lines, corresponding to biological relationships between nodes). Nodes are displayed using shapes as indicated in the key. Nodes marked in red were found to be highly expressed in infected macrophages. Nodes marked in green were found to be highly expressed in uninfected macrophages. Unmarked nodes were added by IPA^® ^due to a high degree of probability of involvement in a given network. The node color intensity is an indication of the degree of up-(red) or down-(green) regulation of genes observed in the biological network analysis from both C57BL/6 and CBA macrophages in response to infection. Solid lines denote direct interactions, whereas dotted lines represent indirect interactions between the genes represented in this network.

IPA^® ^was used to model pathways and networks of the differentially expressed genes by C57BL/6 macrophages in response to *L. amazonensis *infection, in order to infer relationships among these genes by considering their potential involvement in the course and outcome of parasite infection in accordance with host genetic background. To this end, IPA^® ^built the cell morphology and immunological disease network containing 35 genes with the highest probability of being modulated together as a result of infection (score 40, Figure 3A). In this network, 17 genes were down-modulated in infected macrophages, including: *g6pd *(- 2.89), involved in stress oxidative response; *ctcs *(-2.80) which participates in immune response and proteolysis; *sec61b *(-3.03), which participates in protein translocation at the endoplasmic reticulum; *Rab7 *(-2.25), which encodes a small GTPase involved in membrane trafficking during the late endosome maturation process; *Rhogam *(-2.43) known to be involved in cell signaling, adhesion and migration; *vav1 *(-2.49) and *map2k5 *(-2.14) which both encode proteins that participate in cell signaling. Only three genes were found to be up-regulated: *map4k4 *(+2.08), which participates in the ubuquitination process; *tax1bp1 *(+2.12), which encodes a protein involved in proliferation and cellular metabolism; and *arg1 *(+3.16), which encodes arginase 1 (Arg1), known to be involved in cell signaling and stress response.

Very recently, Shweash, M. et al. (2011) showed that *L. mexicana *promastigotes provoke higher levels of Arg1 expression, as well as activation of the MAP kinase-signaling pathway in C57BL/6 macrophages [41]. Additionally, Wilmanski, J. et al. (2007) revealed that the silencing of Map4k4 in macrophages in vivo protected mice from LPS-induced lethality by inhibiting pro-inflammatory molecules, such as TNF-α and interleukin-1β production [42]. Interestingly, these same authors reported that, in comparison with wild-type mice, the glucose-6-phosphate dehydrogenase (G6pd)-deficient mice (g6pd^-/-^) treated with LPS produced greater levels of interleukin (IL)-1β, IL-6, and IL-10 in their sera and peritoneal cavities [42]. These findings are consistent with the data in the present study with respect to the down-regulation of *g6pd *(-2.89) and up-regulation *map4k4 *(+2.08) in infected C57BL/6 macrophages compared to uninfected cells. Taken together, these findings support the notion that the modulation of these genes involved in the host inflammatory response trigger the production of significant amounts of pro-inflammatory cytokines, which is related to the capacity of C57BL/6 macrophages to control *L. amazonensis *infection.

The second network modeled by IPA^® ^was the protein synthesis, cellular development and cell death network (score 38, Figure 3B). This network contains 19 out of the 35 genes that were modulated by C57BL/6 macrophages in response to *L. amazonensis *infection. Most of these genes (14/19) were found to be down-regulated in infected cells, including: *vasp *(-2.06), involved in actin filament organization; *snapin *(-2.28), which participates in intracellular protein transport and exocytosis; *aplp2 *(-2.61) and *rgs19 *(-2.27), which encode proteins from the G protein signaling pathway; *igf1 *(-2.01), involved in cell proliferation and apoptosis; *eef2 *(-2.20), which encodes a protein implicated in transcription processes. A total of five genes (5/19) were up-regulated in infected C57BL/6 macrophages compared to uninfected cells, including: *mt1e *(+9.53), involved in apoptosis and oxidative stress response; *ddx6 *(+2.24), involved in cell replication; *actb *(+1.99), which participates in intracellular transport and endocytosis; *aktip *(+2.21), which encodes a protein that participates in intracellular transport and apoptosis; *adamts1 *(+2.07), involved in an integrin signaling pathway, as well as cellular migration.

In both of the networks modeled by IPA^® ^pertaining to infected C57BL/6 macrophages, namely the cell morphology and immunological disease network, as well as the protein synthesis, cellular development and cell death network, many genes involved in apoptosis were found to be up-regulated. This finding is consistent with the uninfected C57BL/6 macrophage expression profile, which also found up-regulation of genes involved in apoptosis (Figure 3A, B) and is very likely related to the capacity of C57BL/6 macrophages to control parasite infection. This hypothesis is also supported by previous studies which have described the inhibition of apoptosis in host cells using several susceptibility models of *L. donovani *[42,43], as well as *L. major *[44,45] and *L. amazonensis *[22] infection.

#### Genes involved in the lipid metabolism, cellular movement, and small molecule biochemistry network are up-regulated in CBA macrophages in response to *L. amazonensis* infection

Considering *L. amazonensis *infection in CBA macrophages IPA^® ^modeled the lipid metabolism, cellular movement, and small molecule biochemistry network (score 26) containing 35 genes with the highest probability of being modulated together as a result of infection (Figure 3C). Nine out of these 35 genes were found to be up-regulated under infection in CBA cells: *loc340571 *(similar to *hsiah1*, +13.00), *tax1bp1 *(+2.70), *vacuolar H + ATPase, mt1f *(+2.84) and *mt1e *(+5.19), which are all involved in apoptosis, while the latter two are additionally known to play a role in the oxidative stress response; *sf1 *(+2.13), which is implicated in transcriptional regulation and splicing processes; *pla2g4f *(+2.08), which is involved in chemotaxis and cellular migration; *itgav *(+2.30), which participates in cell adhesion; and *eif4g1 *(+2.45), that encodes a protein which participates in translation process regulation.

In accordance with the present findings, the up-regulation of genes involved in the lipid metabolism process has been recently described in BALB/c macrophages [5]. Osorio y Fortéa et al. (2009) suggest that collaborations among these genes likely act to facilitate the survival of *L. amazonensis *inside susceptible macrophages by way of a mechanism involved in the biogenesis of large *L. amazonensis*-induced parasitophorous vacuoles in both BALB/c and CBA macrophages.

#### Comparison of differential gene expression by C57BL/6 and CBA macrophages in response to *L. amazonensis* infection

To gain deeper insight into the differences between the respective responses of C57BL/6 and CBA macrophages to infection, the authors attempted to identify specific genes observed to be significantly modulated in a divergent pattern as a result of *L. amazonensis *infection. However, the baseline gene expression signatures measured prior to infection present a challenge to this type of analysis, as inherent transcriptomic differences may interfere with the accurate identification of differentially expressed gene sets. Firstly, all gene expression values were normalized by subtracting the expression levels by infected macrophages from the corresponding mean expression levels (log_2_-scale) by uninfected cells within a given mouse strain. Thereafter, a direct comparison of normalized gene expression levels was performed using SAM analysis to identify the genes that were differentially expressed between these two mouse strains.

Finally, IPA^® ^was used to highlight possible connections between C57BL/6 and CBA macrophages responses to *L. amazonensis *infection. Networks were constructed from the total number of differentially expressed genes (n = 114), considering both strains of mice. The cell cycle network (See Additional file 6: Figure S2) had the highest probability of interrelated genes being modulated together. This network contains 35 genes (score 36), with 16 out of the 114 genes that were modulated by either C57BL/6 or CBA macrophages in response to *L. amazonensis*. Ten of the 16 modulated genes encode proteins involved in several cellular processes: *usp3*, which encodes an enzyme involved in ubiquitination; *phb *and *polr2a*, which encode proteins implicated in the transcription process; *elf4b*, involved in the translational process; *gstp1*, which participates in detoxification; *rps6ka1 *and *sipa1*, both involved in cellular signaling; *cd72, s1pr2 *and *ptafr*, which encode surface receptors. Of these, *cd72, s1pr2 *and *ptafr *were found to be up-regulated in C57BL/6 macrophages infected with *L. amazonensis *(data not shown). These genes encode receptors, which are expressed on macrophage surfaces. Moreover, the modulation of these receptors and subsequent down-regulation of the macrophage proinflammatory response has been previously described [46,47] and is in accordance with the ability of C57BL/6 macrophages to control *L. amazonensis *infection [3].

Cd72 has been described as a costimulatory molecule found to be up-regulated in macrophages during the activation of a Th1-type immune response [48]. Cd72 participates in the activation of a pro-inflammatory response in the lungs of aging mice and was also found to be associated with an increase in the number of CD4, CD8 and B cells, as well as macrophages. *s1pr2 *encodes the sphingosine-1-phosphate receptor-2 (S1pr2), involved in the recognition of sphingosine-1-phosphate, a biologically active sphingolipid that causes pleiotropic effects in macrophages, and is central to the development of atherosclerosis [48]. Evidence shows that S1pr2 is involved in macrophage retention at the site of atherosclerotic plaque inflammation [49]. The authors suggest further investigation into the role played by both Cd72 and S1pr2 in *L. amazonensis *infection.

The other gene found to be up-regulated in C57BL/6 infected macrophages was *ptafr*, which encodes the receptor for lipid mediator platelet-activating factor (Paf) and is implicated in a number of pathological conditions characterized by tissue inflammation [50]. The role Ptafr plays in protozoan infections has previously been evaluated [51,52]. *Ptafr*^-/- ^mice of C57BL/6 background were found to be more susceptible to infection by *L. amazonensis *than in wild-type controls, as evidenced by both lesion size and parasite number at the site of infection. These findings are associated with the inefficient production of immune mediators, including IFN-γ, Ccl5 and nitric oxide synthase-2 mRNA, as well as being associated with higher levels of arginase-1 mRNA and elevated amounts of antibodies. These authors concluded that signaling through the Ptafr is essential for the murine host to drive an immune response towards controlling *L. amazonensis *infection [53]. The up-regulation of *Ptafr *in *L. amazonensis*-infected C57BL/6 macrophages observed in the present study is consistent with the ability of these cells to control parasite infection, as observed herein.

## Conclusion

In conclusion, the present study represents an initial attempt at making direct comparisons between the global gene expression profiles from two distinct strains of uninfected mouse macrophages. Our analysis revealed that the transcriptional profile of uninfected C57BL/6 macrophages was markedly different from that of CBA macrophages. We also found that C57BL/6 macrophages express higher levels of genes involved in the host immune inflammatory response and apoptosis, as well as others that encode for phagocytic receptors that recognize pathogens and apoptotic cells. These cells were also found to down-regulate genes involved in the deactivation pathway of macrophages. In response to infection, C57BL/6 macrophages continued to up-regulate genes involved in apoptosis, as was similarly observed in uninfected cells. Finally, the authors found a low number of genes, which were related to lipid metabolism, up-regulated by CBA macrophages in response to *L. amazonensis *infection. Collaboration among these genes likely facilitates the survival of *L. amazonensis *inside susceptible macrophages by way of a mechanism involved in the biogenesis of large *L. amazonensis*-induced parasitophorous vacuoles. Taken together, these findings may aid in the understanding of C57BL/6 macrophages' greater capacity to control *L. amazonensis *infection in comparison to CBA cells. However, the mechanism by which these differentially expressed genes affect the course of *Leishmania *infection remains unclear. Further studies should be conducted to investigate the influence of baseline gene expression signatures on the outcome of *L. amazonensis *infection with respect to host genetic background.

## Authors' contributions

CMP performed all bioinformatics and statistical analyses, and drafted the manuscript, RAS performed most of the RT-qPCR experiments, and helped to draft the manuscript, ACD and DPP designed and performed some of the RT-qPCR experiments, JPBM conducted building of the networks using IPA, helped to draft the manuscript, and contributed to the discussion section, TFA and ING carried out the experiments involving tissue culture and RNA extractions, LSO and GAB contributed to the results and discussion section, MAK participated in the design of the study and microarray experiments, PSTV conceived of the study, performed the microarray experiments, participated in the study design and coordination, as well as helped to draft the manuscript. All authors read and approved the final manuscript.

## Supplementary Material

Additional file 1 **Table S1**. Differentially expressed genes in uninfected macrophages from C57BL/6 vs CBA mice. Click here for file

Additional file 2 **Table S2**. Expressed genes in *L. amazonensis*-infected C57BL/6 macrophages. Click here for file

Additional file 3 **Table S3**. Expressed genes in *L. amazonensis*-infected CBA macrophages. Click here for file

Additional file 4 **Table S4**. List of primers used in RT-qPCR amplification of gene expression in uninfected and *L. amazonensis*-infected C57BL/6 and CBA macrophages. Click here for file

Additional file 5 **Figure S1**. Comparative analysis of the kinetics of infection by *L. amazonensis *in C57BL/6 and CBA. C57BL/6 or CBA inflammatory peritoneal macrophages were plated (2 × 10^5^/mL) for 24 h and infected with *L. amazonensis *stationary phase promastigotes at a ratio of 10:1 (parasite to macrophage). After 12 h, cells were washed, reincubated for additional 6 or 24 h and then fixed with ethanol for 20 min. After H&E staining, the percentage of infected cells (**A**) and the parasite numbers per macrophage (**B**) were quantified using light microscopy at each time interval. Results are representative of two independent experiments performed in quadruplicate ± SD. (Mann-Whitney *p = 0.05). Click here for file

Additional file 6 **Figure S2**. Network built using differentially expressed genes in *L. amazonensis*-infected macrophages from C57BL/6 and CBA mice. C57BL/6 and CBA macrophages were cultured separately, then infected and processed for microarray analysis as described in Materials and Methods. The cell cycle network was modeled using IPA^®^. Genes marked in gray represent those found to be differentially expressed between C57BL/6 and CBA infected macrophages, while unmarked genes were added by IPA^® ^due to a high probability of involvement in this network. Similar to Figure 2, the above network is displayed as a series of nodes (genes or gene products) and edges (or lines, corresponding to biological relationships between nodes). Nodes are displayed using shapes as indicated in the key. Solid lines denote direct interactions, whereas dotted lines represent indirect interactions between the genes represented in this network. Click here for file
