# Supplementary material for: A comparison of two distinct murine macrophage gene expression profiles in response to Leishmania amazonensis infection
Source: BMC Microbiol. 2012 Feb 9;12:22. doi: 10.1186/1471-2180-12-22 (PMC3313874; doi:10.1186/1471-2180-12-22)
Supplement: Additional file 1 — Table S1. Differentially expressed genes in uninfected macrophages from C57BL/6 vs CBA mice. [file 1471-2180-12-22-S1.DOC]

Additional file 1

Table S1 - Differentially expressed genes in C57BL/6 vs CBA uninfected macrophages.

| **Gene Symbol** | **Gene Name** | **Function** | **log_2_ FC** | **q-value** |
| --- | --- | --- | --- | --- |
| C1qb | complement component 1, q subcomponent, beta polypeptide | Innate immune response; Complement cascade | +5.081 | 0.000 |
| C1qc | complement component 1, q subcomponent, C chain | Innate immune response; Complement cascade | +5.037 | 0.000 |
| Igsf7 | Immunoglobulin superfamily, member 7 (MAIR II) | Positive regulation of cytokine secretion | +4.864 | 0.000 |
| Gas5 | growth arrest specific 5 | miscRNA; Positive induction of apoptosis | +3.498 | 0.000 |
| Tmem50b | transmembrane protein 50B | ER and Golgi apparatus membrane protein | +3.422 | 0.000 |
| Ctsc | cathepsin C | Lysosomal proteolysis | +3.083 | 0.000 |
| UNK_M74123 | Mus musculus (strain C57BI/6) mRNA sequence | LPS-induced macrophage transcript; Unknown function | +3.037 | 0.000 |
| Chi3l3 | chitinase 3-like 3 | Chitin catabolism; Inflammatory response | +3.028 | 0.000 |
| C1qa | complement component 1, q subcomponent, alpha polypeptide | Innate immune response; Complement cascade | +2.949 | 0.000 |
| Snhg6 | small nucleolar RNA host gene (non-protein coding) 6 | MiscRNA; Unknown function | +2.781 | 0.000 |
| Glb1 | galactosidase, beta 1 | Galactose catabolism; Lysosomal catabolism | +2.753 | 0.000 |
| Apoe | apolipoprotein E | Lipid metabolism; Apoptosis; Pleiotropic | +2.690 | 0.000 |
| Aldh9a1 | aldehyde dehydrogenase 9, subfamily A1 | Carnitine metabolism; Oxidation-reduction; Cellular aldehyde metabolism | +2.539 | 0.000 |
| Apoc2 | apolipoprotein C-II | Lipid metabolism; Lipid transport; Negative regulation of receptor-mediated endocytosis | +2.473 | 0.000 |
| Agtrap | angiotensin II, type I receptor-associated protein | Regulation of blood pressure; Possibly anti-inflammatory; Reduction of macrophage cholesterol efflux | +2.370 | 0.000 |
| Cd5l | CD5 antigen-like | Apoptosis; Receptor-mediated endocytosis | +2.261 | 0.000 |
| Emb | embigin | Cell adhesion | +2.170 | 0.000 |
| Ly9 | lymphocyte antigen 9 | Cell adhesion | +2.133 | 0.000 |
| Prdx2 | peroxiredoxin 2 | Oxidation-reduction; Anti-apoptosis; Negative regulation of NF-kappaB activity; Negative regulation of LPS-mediate signaling; Inflammatory response | +2.046 | 0.000 |
| Xdh | xanthine dehydrogenase | Oxidation-reduction; Xanthine catabolism | +2.025 | 1.438 |
| Cd47 | CD47 antigen (Rh-related antigen, integrin-associated signal transducer) | Cell adhesion; Cell migration; Opsonization; Inflammatory response; Positive regulation of phagocytosis | +2.011 | 1.873 |
| Cd24a | CD24a antigen | Apoptosis; Cell activation; Cell migration; Cell adhesion | +1.996 | 0.000 |
| Mfge8 | milk fat globule-EGF factor 8 protein | Cell adhesion; Phagocytosis | +1.984 | 0.921 |
| Rpl24 | ribosomal protein L24 | Translation; Mitotic G1/S checkpoint | +1.899 | 0.000 |
| Wdr92 | WD repeat domain 92 | Apoptosis; Methylated lysine binding | +1.826 | 0.000 |
| Il10rb | interleukin 10 receptor, beta | Cytokine-cytokine receptor interaction; Jak-STAT signaling | +1.762 | 0.000 |
| Igf1 | insulin-like growth factor 1 | Anti-apoptosis; Insuline-like growth factor receptor signaling | +1.732 | 0.000 |
| Lst1 | leukocyte specific transcript 1 | Negative regulation of lymphocyte proliferation | +1.696 | 0.000 |
| Kif5b | kinesin family member 5B | Vesicle transport along microtubule | +1.667 | 0.000 |
| Gdf15 | growth differentiation factor 15 | Growth factor; Possibly involved with chemotaxis and cell death | +1.511 | 0.000 |
| Psen2 | presenilin 2 | Anti-apoptosis; Apoptosis; Notch signaling | +1.501 | 0.921 |
| Slc9a3r1 | solute carrier family 9 (sodium/hydrogen exchanger), member 3 regulator 1 | Apoptosis; Actin cytoskeleton organization; Wnt receptor signaling; cAMP-mediated signaling; Negative regulation of cell motility, migration and proliferation | +1.450 | 0.000 |
| Htra1 | HtrA serine peptidase 1 | Proteolysis; Negative regulation of BMP signaling; Negative regulation of TGF-beta receptor signaling; Regulation of cell growth | +1.436 | 0.000 |
| Dusp1 | dual specificity phosphatase 1 | Apoptosis; Anti-apoptosis; Inactivation of MAPK | +1.430 | 0.000 |
| Tap1 | transporter 1, ATP-binding cassette, sub-family B (MDR/TAP) | MHC-class I response | +1.427 | 4.336 |
| Ms4a6c | membrane-spanning 4-domains, subfamily A, member 6C | Receptor activity; Unknown function | +1.425 | 4.336 |
| Thbs1 | thrombospondin 1 | Apoptosis; Anti-apoptosis; Inflammation; Cell migration; Cell cycle arrest | +1.409 | 1.438 |
| Pttg1 | pituitary tumor-transforming gene 1 | Cell cycle; DNA repair; Negative regulation of cell proliferation | +1.389 | 0.000 |
| Ifi204 | interferon activated gene 204 | Cellular response to IFN-beta; Negative regulation of transcription | +1.376 | 0.000 |
| Rsad2 | radical S-adenosyl methionine domain containing 2 | Defense response; NF-kappa beta and AP-1 modulator | +1.364 | 0.000 |
| Sod1 | superoxide dismutase 1, soluble | Anti-apoptosis; Oxidation-reduction; Glutathione metabolism | +1.341 | 0.000 |
| Hgsnat | heparan-alpha-glucosaminide N-acetyltransferase | Lysosomal transport; | +1.301 | 0.000 |
| Ptgir | prostaglandin I receptor (IP) | Signal transduction; G-protein coupled receptor signaling | +1.276 | 0.000 |
| 2310008H09Rik | RIKEN cDNA 2310008H09 gene | Unknown function | +1.270 | 0.921 |
| Fcgr1 | Fc receptor, IgG, high affinity I | Innate immune response; Phagocytosis; Antibody-dependent citotoxicity | +1.266 | 0.921 |
| Ccl3 | chemokine (C-C motif) ligand 3 | Inflammation; Chemotaxis | +1.251 | 3.774 |
| Cnpy2 | canopy 2 homolog (zebrafish) | Endoplasmic reticulum protein; Unknown function | +1.223 | 0.000 |
| Adam8 | a disintegrin and metallopeptidase domain 8 | Cell adhesion; Inflammation; Chemotaxis; Positive regulation of NF-kappa beta transcription activity | +1.209 | 0.000 |
| Psmb5 | proteasome (prosome, macropain) subunit, beta type 5 | Proteasomal ubiquitin-dependent proteolysis | +1.209 | 0.000 |
| Spp1 | secreted phosphoprotein 1 | Anti-apoptosis; Cell adhesion | +1.206 | 0.000 |
| Use1 | unconventional SNARE in the ER 1 homolog (S. cerevisiae) | Endoplasmic reticulum to Golgi vesicle-mediated transport | +1.196 | 0.000 |
| Tmem66 | transmembrane protein 66 | Unknown function | +1.189 | 0.000 |
| Fcgr1 | Fc receptor, IgG, high affinity I | Antibody-dependent citotoxicity; Innate immune response; Phagocytosis | +1.184 | 4.866 |
| Tceb3 | transcription elongation factor B (SIII), polypeptide 3 | Regulation of transcription; | +1.183 | 0.000 |
| Aoah | acyloxyacyl hydrolase | Inflammation; LPS metabolism | +1.181 | 4.336 |
| Gadd45b | growth arrest and DNA-damage-inducible 45 beta | Apoptosis; Cell differentiation; Cell cycle regulator | +1.178 | 0.921 |
| Mcfd2 | multiple coagulation factor deficiency 2 | Vesicle-mediated transport | +1.149 | 0.000 |
| Sema4d | sema domain, immunoglobulin domain (Ig), transmembrane domain (TM) and short cytoplasmic domain, (semaphorin) 4D | Cell differentiation | +1.144 | 0.000 |
| Thumpd1 | THUMP domain containing 1 | Unknown function | +1.132 | 0.921 |
| Ddhd1 | DDHD domain containing 1 | Lipid catabolism | +1.129 | 0.000 |
| Fabp4 | fatty acid binding protein 4, adipocyte | Cytokine production; Inflammation; Fatty acid metabolism | +1.114 | 0.000 |
| Cd47 | CD47 antigen (Rh-related antigen, integrin-associated signal transducer) | Cell adhesion; Cell migration; Opsonization; Signal transduction | +1.106 | 3.774 |
| Pdxdc1 | pyridoxal-dependent decarboxylase domain containing 1 | Carboxylic acid metabolism | +1.102 | 0.000 |
| 2310044H10Rik | RIKEN cDNA 2310044H10 gene | Unknown function | +1.066 | 2.387 |
| AI607873 | expressed sequence AI607873 | Unknown function | +1.050 | 0.921 |
| Tsta3 | tissue specific transplantation antigen P35B | Oxidation-reduction; cytolysis; GDP-mannose metabolism | +1.049 | 3.774 |
| Ergic1 | endoplasmic reticulum-golgi intermediate compartment (ERGIC) 1 | Vesicle-mediated transport | +1.047 | 0.000 |
| Tsku | Tsukushin | Unknown function | +1.041 | 3.774 |
| Fam32a | family with sequence similarity 32, member A | Apoptosis | +1.013 | 4.866 |
| Rnase4 | ribonuclease, RNase A family 4 | RNA hydrolysis | +1.013 | 1.873 |
| Cd72 | CD72 antigen | B-cell receptor signaling | +1.007 | 2.387 |
| Bcl2a1a | B-cell leukemia/lymphoma 2 related protein A1a | Apoptosis; B cell homeostasis | +0.996 | 0.000 |
| Napsa | napsin A aspartic peptidase | Proteolysis | +0.990 | 3.774 |
| Ly6e | lymphocyte antigen 6 complex, locus E | Apoptosis | +0.989 | 3.774 |
| Soat1 | sterol O-acyltransferase 1 | Lipid metabolism; Macrophage differentiation | +0.989 | 0.921 |
| Hmgn2 | high mobility group nucleosomal binding domain 2 | Nucleosome binding; Regulation of development | +0.985 | 0.000 |
| Mrpl35 | mitochondrial ribosomal protein L35 | Translation | +0.979 | 3.774 |
| St3gal5 | ST3 beta-galactoside alpha-2,3-sialyltransferase 5 | Protein glycosylation | +0.962 | 0.000 |
| Ifi203 | interferon activated gene 203 | Cellular response to IFN-beta | +0.960 | 0.921 |
| Tmem159 | transmembrane protein 159 | Unknown function | +0.951 | 4.336 |
| Dock2 | dedicator of cyto-kinesis 2 | Chemotaxis; Cytoskeleton organization; T cell activation | +0.945 | 0.000 |
| Ifnar2 | interferon (alpha and beta) receptor 2 | Cell proliferation; Cytokine-mediated signaling; Response to IFN-alpha | +0.942 | 3.774 |
| Pdxdc1 | pyridoxal-dependent decarboxylase domain containing 1 | Carboxylic acid metabolism | +0.938 | 0.000 |
| Tfrc | transferrin receptor | Endocytosis; Proteolysis; | +0.933 | 0.000 |
| Mfhas1 | malignant fibrous histiocytoma amplified sequence 1 | Unknown function | +0.929 | 4.336 |
| Gng2 | guanine nucleotide binding protein (G protein), gamma 2 | G-protein coupled receptor signaling; Cell proliferation | +0.926 | 3.774 |
| LOC216820 | similar to DKFZP566O084 protein | Unknown function | +0.924 | 0.921 |
| Cdc42ep4 | CDC42 effector protein (Rho GTPase binding) 4 | Rho protein signal transduction; Regulation of cell shape | +0.921 | 0.921 |
| Rhoc | ras homolog gene family, member C | Cell cycle cytokinesis; small GTPase mediated signal transduction | +0.915 | 0.000 |
| Srebf1 | sterol regulatory element binding transcription factor 1 | Cellular response to starvation; Insulin receptor signaling; lipid metabolism | +0.914 | 1.873 |
| Zfp68 | zinc finger protein 68 | Unknown function | +0.907 | 2.387 |
| Parp2 | poly (ADP-ribose) polymerase family, member 2 | Base-excision DNA repair | +0.905 | 6.520 |
| Atp6v1e1 | ATPase, H+ transporting, lysosomal V1 subunit E1 | Ion transport; Lysosomal protein | +0.895 | 0.000 |
| Tox4 | TOX high mobility group box family member 4 | DNA binding | +0.888 | 1.873 |
| Npc1 | Niemann Pick type C1 | Autophagy; Endocytosis; Cholesterol homeostasis | +0.877 | 2.387 |
| Arl6ip1 | ADP-ribosylation factor-like 6 interacting protein 1 | Cotranslational protein targeting to membrane | +0.873 | 0.000 |
| Bid | BH3 interacting domain death agonist | Apoptosis; Regulation of G1/S transition | +0.872 | 0.000 |
| Bcl2l11 | BCL2-like 11 (apoptosis facilitator) | Apoptosis; Cell adhesion | +0.867 | 4.336 |
| Uba7 | Ubiquitin-like modifier activating enzyme 7 | ISG15-protein conjugation | +0.861 | 1.438 |
| Rbck1 | RanBP-type and C3HC4-type zinc finger containing 1 | Apoptosis; Protein poliubiquitination; Negative regulator of NF-kappa beta transcription activity | +0.857 | 0.921 |
| Siva1 | SIVA1, apoptosis-inducing factor | Apoptosis; Negative regulator of NF-kappa beta transcription activity | +0.848 | 0.000 |
| Fam105a | family with sequence similarity 105, member A | Unknown function | +0.842 | 4.866 |
| Vps54 | vacuolar protein sorting 54 (yeast) | Growth; Protein transport; Retrograde transport, endosome to Golgi | +0.831 | 4.336 |
| Clcn7 | chloride channel 7 | Ion transport; Response to pH | +0.827 | 0.921 |
| Gcfc1 | GC-rich sequence DNA-binding factor 1 | Unknown function | +0.817 | 1.438 |
| Eif3k | eukaryotic translation initiation factor 3, subunit K | Translation initiation | +0.817 | 0.000 |
| Pcyt2 | phosphate cytidylyltransferase 2, ethanolamine | Phospholipid biosynthesis | +0.815 | 0.921 |
| Ptpn22 | protein tyrosine phosphatase, non-receptor type 22 (lymphoid) | Dephosphorylation; T cell receptor signaling | +0.794 | 3.774 |
| Arl2bp | ADP-ribosylation factor-like 2 binding protein | Maintenance of protein location in nucleus | +0.793 | 1.438 |
| Echs1 | enoyl Coenzyme A hydratase, short chain, 1, mitochondrial | Fatty acid beta-oxidation | +0.793 | 1.438 |
| Entpd1 | ectonucleoside triphosphate diphosphohydrolase 1 | G-protein coupled receptor signaling | +0.788 | 3.774 |
| Grcc10 | gene rich cluster, C10 gene | Unknown function | +0.777 | 1.438 |
| Glb1 | galactosidase, beta 1 | Galactose metabolism; Lysosomal protein | +0.776 | 0.921 |
| Litaf | LPS-induced TN factor | Apoptosis; Positive regulation of NF-kappa beta cascade; Signal transduction | +0.775 | 0.000 |
| Pik3r2 | phosphatidylinositol 3-kinase, regulatory subunit, polypeptide 2 (p85 beta) | Insulin receptor signaling; Regulation of phosphorylation; Signal transduction | +0.768 | 4.336 |
| H2-M3 | histocompatibility 2, M region locus 3 | MHC-class Ib response | +0.767 | 0.000 |
| Igf1 | insulin-like growth factor 1 | Anti-apoptosis; Insulin-like growth factor receptor signaling | +0.766 | 1.438 |
| Use1 | unconventional SNARE in the ER 1 homolog (S. cerevisiae) | Vesicle-mediated transport; Regulation of ER to Golgi vesicle-mediated transport | +0.738 | 0.000 |
| Sdc4 | syndecan 4 | Signal transduction; Positive regulation of focal adhesion assembly; Positive regulation of protein kinase activity | +0.728 | 4.336 |
| Mrpl48 | mitochondrial ribosomal protein L48 | Translation | +0.727 | 1.873 |
| 1810037I17Rik | RIKEN cDNA 1810037I17 gene | Unknown function | +0.719 | 2.387 |
| H2-Eb1 | histocompatibility 2, class II antigen E beta | MHC-class II response | +0.716 | 1.873 |
| Vamp5 | vesicle-associated membrane protein 5 | Vesicle-mediated transport; Cell differentiation | +0.710 | 0.921 |
| Ctsc | cathepsin C | Proteolysis; Lysosomal protein | +0.707 | 0.000 |
| Gna13 | guanine nucleotide binding protein, alpha 13 | G-protein coupled receptor signaling; Rho protein signal transduction; Cell differentiation; Signal transduction | +0.700 | 0.921 |
| 4833420G17Rik | RIKEN cDNA 4833420G17 gene | Unknown function | +0.689 | 0.000 |
| Lilrb3 | leukocyte immunoglobulin-like receptor, subfamily B (with TM and ITIM domains), member 3 | Cytokine-mediated signaling; B cell homeostasis | +0.689 | 1.438 |
| Lgmn | Legumain | Proteolysis; Lysosomal protein | +0.687 | 4.866 |
| Rtn4 | reticulon 4 | Endoplasmic reticulum tubular network organization; Negative regulator of anti-apoptosis; Regulation of cell migration | +0.673 | 6.520 |
| Nsep1 | nuclease sensitive element binding protein 1 | CRD-mediated mRNA stabilization; Negative regulation of apoptosis; Negative regulation of insulin receptor signaling; Positive regulation of cell division | +0.672 | 0.921 |
| Hist1h2bc | histone cluster 1, H2bc | Ununsual histone; Plasminogen receptor in macrophages | +0.670 | 6.520 |
| Cdv3 | carnitine deficiency-associated gene expressed in ventricle 3 | Unknown function | +0.658 | 3.774 |
| Arhgdib | Rho, GDP dissociation inhibitor (GDI) beta | Positive regulation of GTPase activity; Regulation of catalytic activity | +0.658 | 1.873 |
| Ift27 | Intraflagellar transport 27 homolog (Chlamydomonas) | Small GTPase mediated signal transduction | +0.657 | 0.000 |
| Aurkaip1 | aurora kinase A interacting protein 1 | Phosporylation; Positive regulation of proteolysis | +0.656 | 2.387 |
| Qrich1 | glutamine-rich 1 | Unknown function | +0.642 | 0.000 |
| Ndufa3 | NADH dehydrogenase (ubiquinone) 1 alpha subcomplex, 3 | Electron transport chain | +0.640 | 1.438 |
| Cbr1 | carbonyl reductase 1 | Vitamin K metabolism; Oxidation-reduction | +0.638 | 0.921 |
| Aurkaip1 | aurora kinase A interacting protein 1 | Phosporylation; Positive regulation of proteolysis | +0.636 | 0.000 |
| Rsph3a | Radial spoke 3A homolog (Chlamydomonas) | Cilliated cells | +0.632 | 0.000 |
| Glt25d1 | glycosyltransferase 25 domain containing 1 | LPS biosynthesis | +0.625 | 4.866 |
| Hist1h2ba | histone cluster 1, H2ba | Inflammation; Plasminogen activation | +0.616 | 0.921 |
| Pigx | phosphatidylinositol glycan anchor biosynthesis, class X | GPI anchor biosynthesis | +0.616 | 0.000 |
| Gpr65 | G-protein coupled receptor 65 | G-protein coupled receptor signaling; Apoptosis; Actin cytoskeleton reorganization; Signal transduction | +0.616 | 4.336 |
| Gadd45b | growth arrest and DNA-damage-inducible 45 beta | Apoptosis; Activation of MAPKK; Cell differentiation | +0.607 | 0.000 |
| 98369_f_at | NA | Unknown | +0.605 | 1.873 |
| Acat2 | acetyl-Coenzyme A acetyltransferase 2 | Metabolism | +0.604 | 0.921 |
| Atpif1 | ATPase inhibitory factor 1 | Regulation of apoptosis | +0.600 | 0.000 |
| Hal | histidine ammonia lyase | Histidine catabolism | -5.652 | 0.000 |
| Pdxdc1 | pyridoxal-dependent decarboxylase domain containing 1 | Carboxylic acid metabolism | -4.158 | 0.000 |
| Cadm1 | cell adhesion molecule 1 | Apoptosis; Cell adhesion; Positive regulation of cytokine secretion | -3.838 | 0.000 |
| Ifi202b | interferon activated gene 202B | Cellular response to IFN-beta | -3.686 | 0.000 |
| Cadm1 | cell adhesion molecule 1 | Apoptosis; Cell adhesion; Positive regulation of cytokine secretion | -3.245 | 0.000 |
| Maoa | monoamine oxidase A | Dopamine catabolism; Oxidation-reduction | -3.079 | 0.000 |
| Mela | melanoma antigen | Unknown function | -3.077 | 6.520 |
| Chi3l1 | chitinase 3-like 1 | Chitin catabolism | -2.868 | 0.771 |
| Ccr5 | chemokine (C-C motif) receptor 5 | G-protein coupled receptor signaling; Response to LPS; Positive regulation of cell-cell adhesion; Negative regulation of macrophage apoptosis; Positive regulation of inflammation | -2.520 | 0.000 |
| 2810025M15Rik | RIKEN cDNA 2810025M15 gene | Unknown function | -2.157 | 0.000 |
| Ckb | creatine kinase, brain | Phosporylation; Cellular chloride ion homeostasis | -2.043 | 0.771 |
| Tor3a | torsin family 3, member A | Chaperone mediated protein folding requiring cofactor | -2.028 | 0.000 |
| Mt2 | metallothionein 2 | Cellular zinc ion homeostatis; Nitric oxide mediated signal transduction | -1.912 | 0.771 |
| Ocel1 | occludin/ELL domain containing 1 | Unknown function | -1.864 | 0.000 |
| Glo1 | glyoxalase 1 | Anti-apoptosis; Glutathione metabolism | -1.855 | 1.346 |
| Gng10 | guanine nucleotide binding protein (G protein), gamma 10 | G-protein coupled receptor signaling; Signal transduction | -1.766 | 0.000 |
| Dusp6 | dual specificity phosphatase 6 | Inactivation of MAPK; Negative regulation of ERK1/ERK2 cascade; Positive regulation of apoptosis | -1.661 | 4.866 |
| Cap1 | CAP, adenylate cyclase-associated protein 1 (yeast) | Actin cytoskeleton organization; Cell morphogenesis; Receptor-mediated endocytosis | -1.659 | 0.000 |
| NA | histocompatibility 2, D region locus 1 | MHC-class II response | -1.634 | 0.000 |
| Ptpra | protein tyrosine phosphatase, receptor type, A | Insulin receptor signaling | -1.617 | 0.000 |
| Apobec1 | apolipoprotein B mRNA editing enzyme, catalytic polypeptide 1 | LPS biosynthesis; mRNA modification; Defense response | -1.496 | 0.000 |
| Ssr1 | signal sequence receptor, alpha | Protein processing in ER | -1.489 | 0.771 |
| Alad | aminolevulinate, delta-, dehydratase | Heme biosynthesis | -1.449 | 0.771 |
| Eps8 | epidermal growth factor receptor pathway substrate 8 | Actin cytoskeleton | -1.440 | 2.387 |
| Il10ra | interleukin 10 receptor, alpha | Cytokine-cytokine receptor interaction; Jak-STAT signaling | -1.391 | 0.000 |
| LOC67527 | murine leukemia retrovirus | Unknown function | -1.319 | 1.346 |
| Ifi202b | interferon activated gene 202B | Cellular response to IFN-beta | -1.309 | 2.387 |
| Eno2 | enolase 2, gamma neuronal | Glycolysis/gluconeogenesis | -1.249 | 6.520 |
| Pltp | phospholipid transfer protein | Lipid transport | -1.191 | 0.771 |
| Ctse | cathepsin E | MHC-class II response; Proteolysis | -1.139 | 1.873 |
| Ngrn | neugrin, neurite outgrowth associated | Cell differentiation | -1.134 | 3.774 |
| Ankrd33b | ankyrin repeat domain 33B | DNA binding | -1.114 | 1.346 |
| Acvrl1 | activin A receptor, type II-like 1 | Negative regulation of cell adhesion, cell growth, cell migration; Positive regulation of BMP signaling | -1.070 | 6.520 |
| Rab10 | RAB10, member RAS oncogene family | Small GTPase mediated signal transduction; Intracellular protein transport | -1.057 | 0.771 |
| Ncf4 | neutrophil cytosolic factor 4 | Cell communication | -1.032 | 1.873 |
| Ppap2b | phosphatidic acid phosphatase type 2B | Cell-cell adhesion; Protein stabilization; Wnt receptor signaling | -1.030 | 1.346 |
| Ctnnb1 | catenin (cadherin associated protein), beta 1 | Apoptosis; Wnt receptor signaling; Cell adhesion | -1.014 | 0.000 |
| Mt1 | metallothionein 1 | Nitric oxide mediated signal transduction; Cellular zinc ion homeostasis | -0.991 | 1.873 |
| Galnt11 | UDP-N-acetyl-alpha-D-galactosamine:polypeptide N-acetylgalactosaminyltransferase 11 | Metabolism | -0.931 | 0.771 |
| Slc9a1 | solute carrier family 9 (sodium/hydrogen exchanger), member 1 | Ion transport; Positive regulation of anti-apoptosis | -0.921 | 1.346 |
| Arhgap39 | Rho GTPase activating protein 39 | Rho-GTPase signaling | -0.888 | 4.866 |
| Bckdhb | branched chain ketoacid dehydrogenase E1, beta polypeptide | Oxidation-reduction; Branched chain family amino acid catabolism | -0.851 | 1.873 |
| Pold3 | polymerase (DNA-directed), delta 3, accessory subunit | Nucleotide-excision repair; DNA replication | -0.847 | 4.336 |
| Fah | fumarylacetoacetate hydrolase | Arginine, tyrosine and L-phenylalanine catabolism; | -0.844 | 6.520 |
| Ctsl | cathepsin L | Cell communication; Proteolysis | -0.840 | 0.000 |
| Gipc1 | GIPC PDZ domain containing family, member 1 | G-protein coupled receptor signaling; Regulation of protein stability | -0.803 | 0.000 |
| Pbx2 | pre B-cell leukemia transcription factor 2 | Positive regulation of transcription | -0.774 | 2.387 |
| Plod3 | procollagen-lysine, 2-oxoglutarate 5-dioxygenase 3 | Collagen fibril organization; Protein localization | -0.764 | 0.000 |
| Pi4ka | phosphatidylinositol 4-kinase, catalytic, alpha polypeptide | Phosphatidylinositol phosphorylation | -0.760 | 0.771 |
| Idh2 | isocitrate dehydrogenase 2 (NADP+), mitochondrial | Oxidation-reduction; TCA cycle | -0.744 | 1.346 |
| Slc44a1 | solute carrier family 44, member 1 | Choline transport | -0.708 | 0.771 |
| Rhob | ras homolog gene family, member B | Apoptosis; Cell adhesion; Protein transport | -0.683 | 2.387 |
| Cadm1 | cell adhesion molecule 1 | Apoptosis; Cell adhesion; Positive regulation of cytokine secretion | -0.677 | 0.000 |
| Cxcl14 | chemokine (C-X-C motif) ligand 14 | Immune response | -0.648 | 0.771 |
| Txndc5 | thioredoxin domain containing 5 | Cell redox homeostasis | -0.646 | 4.866 |
| Spryd4 | SPRY domain containing 4 | Unknown function | -0.641 | 6.520 |
| Itpk1 | inositol 1,3,4-triphosphate 5/6 kinase | Inositol triphosphate metabolism | -0.637 | 6.520 |
| Gpc1 | glypican 1- | Heparan sulfate proteoglycan binding | -0.636 | 1.346 |
| Lasp1 | LIM and SH3 protein 1 | Ion transport | -0.627 | 4.866 |
| Clec16a | C-type lectin domain family 16, member A | Unknown function | -0.601 | 3.774 |
