# Supplementary material for: A comparison of two distinct murine macrophage gene expression profiles in response to Leishmania amazonensis infection
Source: BMC Microbiol. 2012 Feb 9;12:22. doi: 10.1186/1471-2180-12-22 (PMC3313874; doi:10.1186/1471-2180-12-22)
Supplement: Additional file 2 — Table S2. Expressed genes in L. amazonensis-infected C57BL/6 macrophages. [file 1471-2180-12-22-S2.DOC]

Additional file 2

Table S2 – Differentially expressed genes in *L. amazonensis*-infected C57BL/6 macrophages.

| **Gene Symbol** | **Gene Name** | **Function** | **log2 FC** |
| --- | --- | --- | --- |
| Mt1e | metallothionein 1E | Cellular metal ion homeostasis; Nitric oxide mediated signaling; Lysosomal protein | 9.534 |
| Arg1 | arginase, liver | Arginine metabolism; Positive regulation of endothelial cell proliferation | 3.155 |
| Derl2 | Der1-like domain family, member 2 | Response to unfolded protein; Positive regulation of cell growth | 2.285 |
| Ddx6 | DEAD box polypeptide 6 | RNA degradation; Cytoplasm mRNA processing body | 2.238 |
| Svs6 | seminal vesicle secretory protein 6 | Unknown function | 2.235 |
| Aktip | Thymoma viral proto-oncogene 1 interacting protein | Apoptosis; Endosome to lysosome transport; Protein transport | 2.206 |
| Flg | Filaggrin | Cytoskeleton organization | 2.164 |
| Otud4 | OTU domain containing 4 | Unknown function | 2.141 |
| Hcg 1787519 | ribosomal protein L36a pseudogene 8 | Translation | 2.130 |
| Tax1bp1 | Tax1 (human T-cell leukemia virus type I) binding protein 1 | Apoptosis; Anti-apoptosis; Negative regulation of NF-kappaB transcription factor activity | 2.124 |
| Brd7 | bromodomain containing 7 | Cell cycle; Wnt receptor signaling; Regulation of transcription | 2.083 |
| Map4k4 | mitogen-activated protein kinase kinase kinase kinase 4 | Regulation of JNK cascade; Response to stress | 2.083 |
| Adamts1 | ADAM metallopeptidase with thrombospondin type 1 motif, 1 | Metabolism; Proteolysis; Extracellular matrix protein | 2.068 |
| Cnot7 | CCR4-NOT transcription complex, subunit 7 | Regulation of transcription; CCR4-NOT complex | 2.053 |
| Grhpr | glyoxylate reductase/hydroxypyruvate reductase | Oxidation-reduction; Dicarboxylic acid metabolism | 1.996 |
| Actb | actin, beta | Cytoskeleton; Phagosome | 1.986 |
| Hla-b | major histocompatibility complex, class I, B | MHC-class I response | -4.485 |
| Cotl1 | coactosin-like 1 | Cytoskeleton; Defense response | -3.082 |
| Sec61b | Sec61 beta subunit | Protein transport; Retrograde protein transport, ER to cytosol | -3.031 |
| Snx2 | sorting nexin 2 | Cell communication; Intracellular protein transport | -2.961 |
| G6pd | glucose-6-phosphate dehydrogenase | Oxidation-reduction; Cytokine production; Pentose biosynthesis | -2.888 |
| Ubl5 | ubiquitin-like 5 | Protein modification | -2.822 |
| Ccdc56 | coiled-coil domain containing 56 | Mitochondrial membrane | -2.799 |
| Ctsc | cathepsin C | Proteolysis; Lysosomal protein | -2.795 |
| Isyna1 | inositol-3-phosphate synthase 1 | Inositol biosynthesis | -2.753 |
| Ten1 | Telomerase capping complex subunit homolog (S. cerevisiae) | Telomere; DNA binding | -2.746 |
| Nans | N-acetylneuraminic acid synthase | Amino and nucleotide sugar metabolism | -2.632 |
| Aplp2 | amyloid beta (A4) precursor-like protein 2 | Celullar copper ion homeostasis; Extracellular matrix organization; Regulation of EGF-activated receptor activity | -2.612 |
| Gnpda1 | glucosamine-6-phosphate deaminase 1 | Glucosamine catabolism | -2.558 |
| Hsd17b10 | hydroxysteroid (17-beta) dehydrogenase 10 | Oxidation-reduction | -2.548 |
| C19orf60 | chromosome 19 open reading frame 60 | Unknown function | -2.547 |
| Imp3 | IMP3, U3 small nucleolar ribonucleoprotein, homolog | rRNA processing | -2.506 |
| Sirpa | signal-regulatory protein alpha | Phagocytosis; Actin filament organization; Cell-matrix adhesion | -2.504 |
| Flii | flightless I homolog | Actin cytoskeleton organization; Regulation of transcription | -2.497 |
| Vav1 | vav 1 guanine nucleotide exchange factor | Phagocytosis; Integrin-mediated signaling; T cell activation; Regulation of Rho protein signaling | -2.490 |
| Sep15 | Selenoprotein | Oxidation-reduction | -2.475 |
| Stxbp2 | syntaxin binding protein 2 | Exocytosis; Vesicle-mediated transport | -2.470 |
| Sigmar1 | sigma non-opioid intracellular receptor 1 | Ergosterol biosynthesis; Lipid transport | -2.468 |
| Mrpl4 | mitochondrial ribosomal protein L4 | Translation | -2.434 |
| Rhog | ras homolog gene family, member G | Cell chemotaxis; Actin cytoskeleton organization; Rac and Rho protein signaling | -2.425 |
| Cct3 | chaperonin containing TCP1, subunit 3 (gamma) | Protein folding | -2.397 |
| Tmed2 | transmembrane emp24 domain trafficking protein 2 | Golgi organization; Intracellular protein transport | -2.351 |
| Fxc1 | fracture callus 1 homolog | Protein import into mitochondrial inner membrane | -2.342 |
| Commd4 | COMM domain containing 4 | Unknown function | -2.340 |
| Tmem205 | transmembrane protein 205 | Membrane protein | -2.335 |
| Rbck1 | RanBP-type and C3HC4-type zinc finger containing 1 | Positive regulation of apoptosis; Proteasomal ubiquitin-dependent protein catabolism | -2.308 |
| Pea15 | phosphoprotein enriched in astrocytes 15 | Apoptosis; Anti-apoptosis; Carbohydrate transport | -2.291 |
| Atp13a2 | ATPase type 13A2 | Cation transport; Lysosomal protein | -2.284 |
| C9orf16 | chromosome 9 open reading frame 16 | Unknown function | -2.279 |
| Snapin | SNAP-associated protein | Exocytosis | -2.278 |
| Mrps34 | mitochondrial ribosomal protein S34 | Translation | -2.267 |
| Rgs19 | regulator of G-protein signaling 19 | Negative regulation of signal transduction | -2.267 |
| Dctn3 | dynactin 3 (p22) | Cell cycle; Cytokinesis | -2.261 |
| Tcea1 | transcription elongation factor A (SII), 1 | Positive regulation of transcription | -2.261 |
| Fbxo6 | F-box protein 6 | DNA repair; ER-associated protein catabolism; SCF-dependent proteasomal ubiquitin-dependent protein catabolism | -2.257 |
| Arl8b | ADP-ribosylation factor-like 8B | Cell cycle; Mitosis; Small GTPase mediated signal transduction | -2.251 |
| Rab7 | RAB7, member RAS oncogene family | Endosome to lysosome transport; EGF catabolism; Small GTPase mediated signaling | -2.247 |
| Bre | brain and reproductive organ-expressed | Apoptosis; Double-strand break repair | -2.243 |
| Clcn7 | chloride channel 7 | Ion transport; Lysosomal protein | -2.237 |
| Bin1 | bridging integrator 1 | Cell differentiation; Endocytosis; Positive regulation of GTPase activity | -2.216 |
| Fads1 | fatty acid desaturase 1 | Oxidation-reduction; Arachidonic acid metabolism; Lipid biosynthesis | -2.212 |
| Eef2 | eukaryotic translation elongation factor 2 | Translation | -2.204 |
| C19orf20 | chromosome 19 open reading frame 20 | Cell differentiation; Centrosome; Cytoskeleton | -2.181 |
| Adss | adenylosuccinate synthase | Purine metabolism; Aspartate metabolism | -2.169 |
| Rab3d | RAB3D, member RAS oncogene family | Exocytosis; Small GTPase mediated signaling | -2.159 |
| Hagh | hydroxyacylglutathione hydrolase | Glutathione metabolism | -2.151 |
| Edem2 | ER degradation enhancer, mannosidase alpha-like 2 | Endoplasmic reticulum | -2.145 |
| Map2k5 | mitogen-activated protein kinase kinase 5 | Positive regulation of cell growth; BMK cascade; MAPKKK cascade | -2.139 |
| Cox4nb | COX4 neighbor | Unknown function | -2.135 |
| Ndufb2 | NADH dehydrogenase (ubiquinone) 1 beta subcomplex, 2 | Electron transport chain | -2.132 |
| Slc25a6 | solute carrier family 25 (mitochondrial carrier; adenine nucleotide translocator), member 6 | S-adenosylmethionine transport | -2.125 |
| Mat2a | methionine adenosyltransferase II, alpha | S-adenosylmethionine biosynthesis; Circadian rhythm; one-carbon metabolism | -2.124 |
| Ndufv1 | NADH dehydrogenase (ubiquinone) flavoprotein 1 | Electron transport chain; Oxidation-reduction | -2.107 |
| Taf9 | TAF9 RNA polymerase II, TATA box binding protein (TBP)-associated factor | Negative regulation of apoptosis; Histone H3 acetylation; Positive regulation of response to cytokine stimulus | -2.093 |
| Ext2 | exostosin 2 | Cell differentiation; Glycosaminoglycan biosynthesis | -2.080 |
| Kiaa0247 | KIAA0247 | Membrane protein | -2.079 |
| Scarb1 | scavenger receptor class B, member 1 | Cell adhesion; Cholesterol homeostasis; Positive regulation of nitric-oxide synthase activity | -2.071 |
| Lats2 | LATS, large tumor suppressor, homolog 2 | G1/S transition; Negative regulation of canonical Wnt receptor signaling | -2.069 |
| Ufm1 | ubiquitin-fold modifier 1 | Protein ufmylation | -2.067 |
| Znf187 | zinc finger protein 187 | Regulation of transcription; Viral reproduction | -2.065 |
| Vasp | vasodilator-stimulated phosphoprotein | Actin cytoskeleton organization | -2.064 |
| Gps1 | G protein pathway suppressor 1 | Cullin deneddylation | -2.060 |
| Atxn10 | ataxin 10 | Protein binding | -2.056 |
| Rpp21 | ribonuclease P/MRP subunit | tRNA processing | -2.055 |
| Lage3 | L antigen family, member 3 | Unknown function | -2.052 |
| Aldh2 | aldehyde dehydrogenase 2 family (mitochondrial) | Oxidation-reduction; Metabolism | -2.036 |
| Tiprl | TIP41, TOR signaling pathway regulator-like | DNA damage checkpoint; Negative regulation of protein phosphatase type 2A activity | -2.034 |
| Ptdss1 | phosphatidylserine synthase 1 | Phosphatidylserine biosynthesis | -2.016 |
| Ptov1 | prostate tumor overexpressed 1 | Regulation of transcription | -2.014 |
| Rps6ka1 | ribosomal protein S6 kinase, polypeptide 1 | Negative regulation of apoptosis; Intracellular protein kinase cascade; Positive regulation of transcription | -2.014 |
| Nde1 | nudE nuclear distribution gene E homolog 1 | Cell cycle; Cell differentiation; Vesicle transport along microtubule | -2.009 |
| Igf1 | insulin-like growth factor 1 (somatomedin C) | Anti-apoptosis; Insuline-like growth factor receptor signaling | -2.007 |
| Ndufb10 | NADH dehydrogenase (ubiquinone) 1 beta subcomplex, 10 | Electron transport chain | -2.006 |
| Ctps2 | CTP synthase II | Glutamine metabolism; Mitochondrion | -2.003 |
| Samm50 | sorting and assembly machinery component 50 homolog | Protein import into mitochondrial outer membrane | -1.989 |
| Csnk1g2 | casein kinase 1, gamma 2 | Wnt receptor signaling; Protein phosphorylation | -1.987 |
| Gm3258 | predicted gene 3258 | Positive regulation of transcription elongation | -1.984 |
| Erh | enhancer of rudimentary homolog | Unknown function | -1.982 |
| Polr2e | polymerase (RNA) II (DNA directed) polypeptide E | Transcription | -1.974 |
| Xbp1 | X-box binding protein 1 | ER unfolded protein response; regulation of transcription | -1.974 |
| Nop10 | NOP10 ribonucleoprotein homolog (yeast) | rRNA processing | -1.969 |
| Mtm1 | myotubularin 1 | Endosome to lysosome transport; Mitochondrion distribution; Protein dephosphorylation | -1.966 |
| Fam89b | family with sequence similarity 89, member B | Unknown function | -1.964 |
| Pgls | 6-phosphogluconolactonase | Pentose-phosphate shunt | -1.964 |
| Mid1ip1 | MID1 interacting protein 1 (gastrulation specific G12 homolog) | Lipid biosynthesis; Negative regulation of microtubule depolymerization | -1.961 |
