# Supplementary material for: A comparison of two distinct murine macrophage gene expression profiles in response to Leishmania amazonensis infection
Source: BMC Microbiol. 2012 Feb 9;12:22. doi: 10.1186/1471-2180-12-22 (PMC3313874; doi:10.1186/1471-2180-12-22)
Supplement: Additional file 3 — Table S3. Expressed genes in L. amazonensis-infected CBA macrophages. [file 1471-2180-12-22-S3.DOC]

Additional file 3

Table S3 – Up-regulated genes in *L. amazonensis*-infected CBA macrophages

| **Gene Symbol** | **Gene Name** | **Function** | **log2 FC** |
| --- | --- | --- | --- |
| Loc340571 | seven in absentia homolog 1-like | Apoptosis; Cell cycle; Protein ubiquitination | 12.997 |
| Mt1e | metallothionein 1e | Cellular zinc ion homeostasis | 5.194 |
| Mt1f | metallothionein 1f | Cellular zinc ion homeostasis | 2.838 |
| Tax1bp1 | Tax1 (human T-cell leukemia virus type I) binding protein 1 | Anti-apoptosis; Apoptosis; Negative regulation of NF-kappa beta transcription factor activity | 2.690 |
| Eif4g1 | eukaryotic translation initiation factor 4 gamma, 1 | Translation initiation | 2.452 |
| Itgav | integrin, alpha V (vitronectin receptor, alpha polypeptide, antigen CD51) | Cell adhesion; Cell migration; Cell differentiation; Inflammation; Negative regulation of apoptosis | 2.297 |
| Atp6ap1 | ATPase, H+ transporting, lysosomal accessory protein 1 | Ion transport; Cell death; Lysosomal protein | 2.156 |
| Sf1 | splicing factor 1 | RNA splicing; Regulation of steroid biosynthesis | 2.128 |
| Pla2g4f | phospholipase A2, group IVF | Prostaglandin biosynthesis | 2.078 |
