# Supplementary material for: A comparison of two distinct murine macrophage gene expression profiles in response to Leishmania amazonensis infection
Source: BMC Microbiol. 2012 Feb 9;12:22. doi: 10.1186/1471-2180-12-22 (PMC3313874; doi:10.1186/1471-2180-12-22)
Supplement: Additional file 4 — Table S4. List of primers used in RT-qPCR amplification of gene expression in uninfected and L. amazonensis-infected C57BL/6 and CBA macrophages. [file 1471-2180-12-22-S4.DOC]

Additional file 4

Table S4. List of primers used in RT-qPCR amplification of gene expression in uninfected and *L. amazonensis*-infected C57BL/6 and CBA macrophages

| **Gene** | **Forward** | **Reverse** | **Tm (ºC)** |
| --- | --- | --- | --- |
| *Agtrap* | CCGTGAACGAGGGGGTGAGC | TCTGTGATAGGGAATGGCGGATGC | 55 |
| *Apoc2* | TCCAGGGGAACCAGGAAGATG | GTAAAAATGCCTGCGTAAGTGCTC | 55 |
| *Apoe* | CTCGAGTGGCAAAGCAACC | TCCGTCATAGTGTCCTCCATCAGT | 55 |
| *Atox1* | CTGTGAGGGCTGTGCTGAAG | TGGGGCCAAGGTAGGAAACA | 55 |
| *Atp6v1e1* | GAAAGCCGGCTGGACCTGATAG | GCCGTGGGGGAACTGCTC | 55 |
| *Bre* | CAAAACTTACTCTGCTGCTGATGTG | AGACTGACTGAAATGTGAGCGTAGG | 60 |
| *C1qa* | ATGACCCTAGTATGGACAGTGG | CCTTTAAAACCTCGGATACC | 55 |
| *C1qb* | CTACGGGGCTACACAGAAAGTCG | CCGGGAGCTGGCATGATAGGTGA | 55 |
| *Ccl3* | CCCGACTGCCTGCTGCTTCT | GATCTGCCGGTTTCTCTTAGTCA | 55 |
| *Ccr5* | TCATAGAGGGCCCAAGATACAT | TTCCCGGCCCTGATAAAAG | 55 |
| *Cd24a* | TGGGCAGAGCGATGGTG | CGGGAAACGGTGCAACAGATG | 55 |
| *Cd47* | AGTGCACGGCCCCCTTTTG | TCCGTCACTTCCCTTCACCTATTC | 55 |
| *Cdl5* | CCCCCAAAACCCGGAAAATCTAT | TCGAGGCTGAGGGAAAGGTGTCTA | 55 |
| *Ckb* | CCACCTGGGGAAGCACGAGAAGT | GACATCAAACACCCCACCGACAGC | 55 |
| *Ctnnb1* | TGAGGACAAGCCACAGGATTA | CCAGAGTGAAAAGAACGGTAGC | 55 |
| *Ctsc* | GGTTGTATCTTGCAGCCCCTATG | TTCTTCCACCACCCCAAAATCTT | 60 |
| *Emb* | CCGCACAGGTTCCCATTG | TCTGCTCCTCACTCTCCCCTAACT | 55 |
| *Gapdh* | CGACTTCAACAGCAACTCCCACTC | CACCCTGTTGCTGTAGCCGTATTC | 60 |
| *Ergic* | CTACAGCCACACGGGACGCATTAT | GATCTTCTTCCACGCTTCTGAGG | 55 |
| *Gng10* | GTCTTCCGGGGCCAGCGTGAGC | GGCGTCCTTGCATGCATTCTGTAT | 55 |
| *Ifnarf* | GTTTTCGTGAGCACTATCGTAATG | CAGTCTCTTCTTCTTGTTTGTGG | 55 |
| *Ifi202b* | CCACAAAGGTCCCAAACAAGT | TGCCAGAGACCCATACATACCA | 55 |
| *Ifi203* | AATGGCAGTGGTGGTTTAT | GAGTGGCTTTCCTTCATTG | 55 |
| *Ifi204* | TGGGTGCAATGGGTTTCTG | ATCATCTCCAATTCCATAGTAA | 55 |
| *Igsf4a* | TCATCATTCTGGGCCGCTATTTTG | CGTCATCGGCTCCTTTGGCTTCAT | 55 |
| *Il10r* | CTCTTCCTCCTGGGCTGCTTTGTC | TCTTCGCTGATGATGCTTAGTTTG | 55 |
| *Itgav* | AGGGCTGCTGCTACTGGCTGTGTT | ACGGTGGTGAACTTGGAGCGGACAG | 70 |
| *Litaf* | ACCGCCCCGTCCAGATGTG | CTACGCAGAACGGGATGAAGC | 55 |
| *Loc340571* | TGGGTGATGATGCAGTCTTGTTTT | TCAGCTTGTTTGCGTGTTCCTATC | 65 |
| *Map4k4* | GCACAAAAGGGCTCAGAGACTAAAG | CCACCAGAGCGGACGGAGGCAAAGA | 60 |
| *Mt2* | GCGTGTGCTGGCCATATCCCTTGAG | GCCGCCTGCACACGCCCTTTTTCTG | 60 |
| *Ncf4* | AGGATGCTGAGGGGGACTTG | GGCCAGGAGGTTTGCATCTTACAT | 55 |
| *Ndufb10* | CGCTCCCTAACCCCATCACCTACTT | GTTCGGTTCTTGGCATGCTGTCGTT | 70 |
| *Ndufv1* | CCGATTTGTGAAGGGAGATGC | CAAGCTCTGGCCTGAAATGTC | 60 |
| *Ocel1* | CGCTGCCCCCGCCACGAA | GGAGGAGGAGCCCACCACCATCT | 55 |
| *Prdx2* | TTGCTTACAGGGGTCTCTTTATCA | CTTCAGGCTCACCCATGTTTACCC | 55 |
| *Rab7a* | ATTGCTGTGTTCTGGTGTTTGATGT | AGCCTGTGCCCTCTTTGTGG | 60 |
| *Rab10* | GCAAGGGAGCATGGTATTAGGTTT | GATGGCAGCAGATGGACAGGAGAG | 55 |
| *Rgs19* | AGGCGCGACTTATCTATGAGGAC | CGGTAGGTGGGGGAGGTAAGGAATC | 60 |
| *Rhoc* | TGTCCCAACGTGCCCATCATCC | CCTCCCGCACCCCTTCCTTAGTCT | 55 |
| *Rhog* | GGGAGGGCACCAGGTCACTACTT | GCCCCATCGCCCACCACCA | 60 |
| *Sdc4* | CCCAGGGCAGCAACATCTT | ACTGCCTTCGTCCTTCTTCTTCAT | 55 |
| *Sf1* | GGCTCCGACCCCCACCCACAT | GCCCGACAAGCAGGATTAGTTAGC | 60 |
| *Snapin* | TGCTTAATGCCAGGCGACGAGTTGT | CCCGTGCTTATTTGCTTGGAGAACC | 60 |
| *Snx2* | GGAGGCGAAAGTACAACAAGGAGAG | TCACAGGGGTAAGGCTAGGCAATGG | 60 |
| *Ssp1* | TCCCCAACGGCCGAGGTGATAGC | CTGCCCTTTCCGTTGTTGTCCTGA | 55 |
| *Tax1bp1* | CTTAGCCGGCCGGATGGTTTAGAG | GCTCGCTGCACATTGGACACACC | 60 |
| *Vav1* | CGGATCGGCTGGTTCCCTTCTAA | CATGGCCCCTCAACCCTGCTA | 60 |
