# Supplementary figures and images for: A comparison of two distinct murine macrophage gene expression profiles in response to Leishmania amazonensis infection
Source: BMC Microbiol. 2012 Feb 9;12:22. doi: 10.1186/1471-2180-12-22 (PMC3313874; doi:10.1186/1471-2180-12-22)

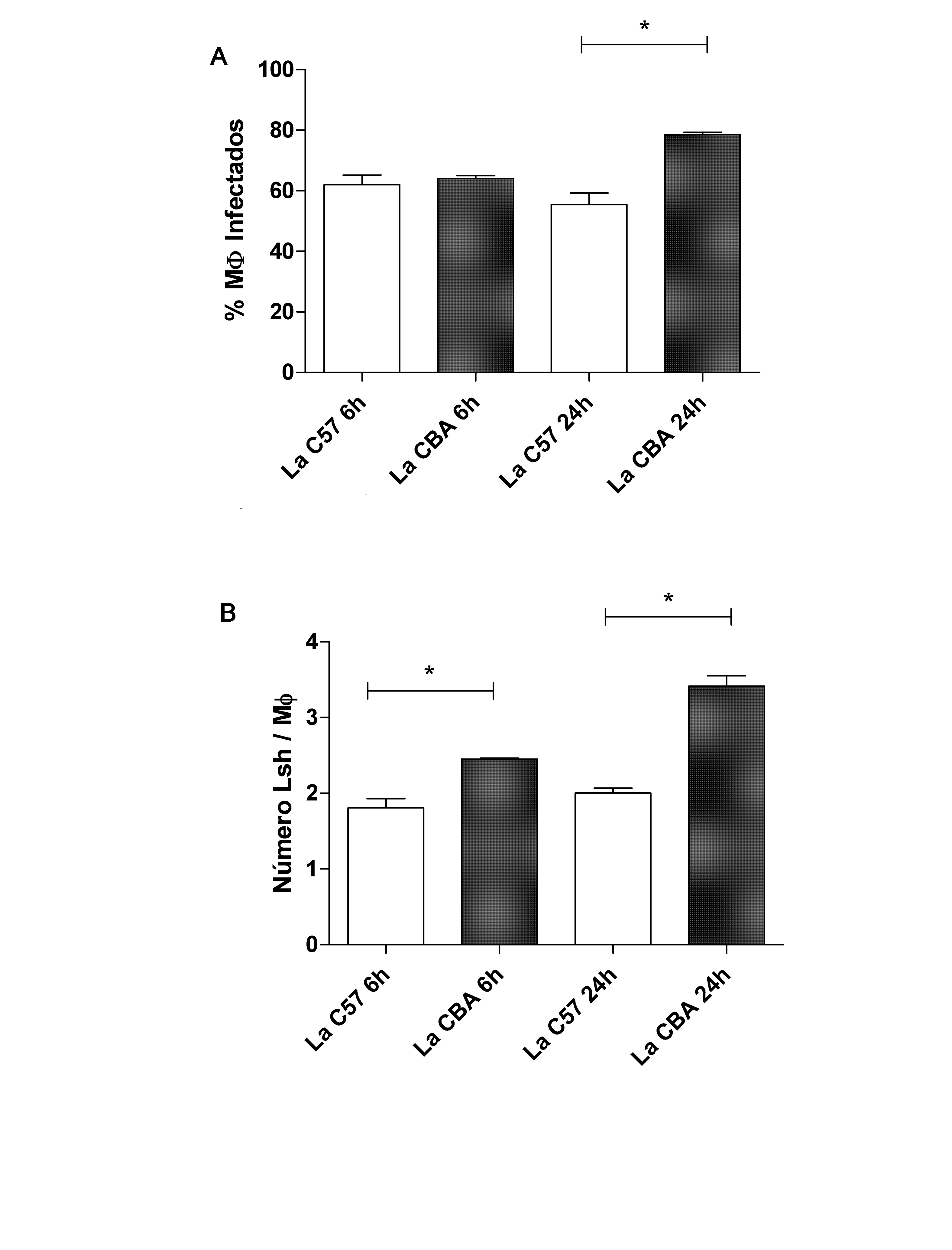

Supplement: Additional file 5 — Figure S1. Comparative analysis of the kinetics of infection by L. amazonensis in C57BL/6 and CBA. C57BL/6 or CBA inflammatory peritoneal macrophages were plated (2 × 10^5^/mL) for 24 h and infected with L. amazonensis stationary phase promastigotes at a ratio of 10:1 (parasite to macrophage). After 12 h, cells were washed, reincubated for additional 6 or 24 h and then fixed with ethanol for 20 min. After H&E staining, the percentage of infected cells (A) and the parasite numbers per macrophage (B) were quantified using light microscopy at each time interval. Results are representative of two independent experiments performed in quadruplicate ± SD. (Mann-Whitney *p = 0.05). [file 1471-2180-12-22-S5.TIFF]

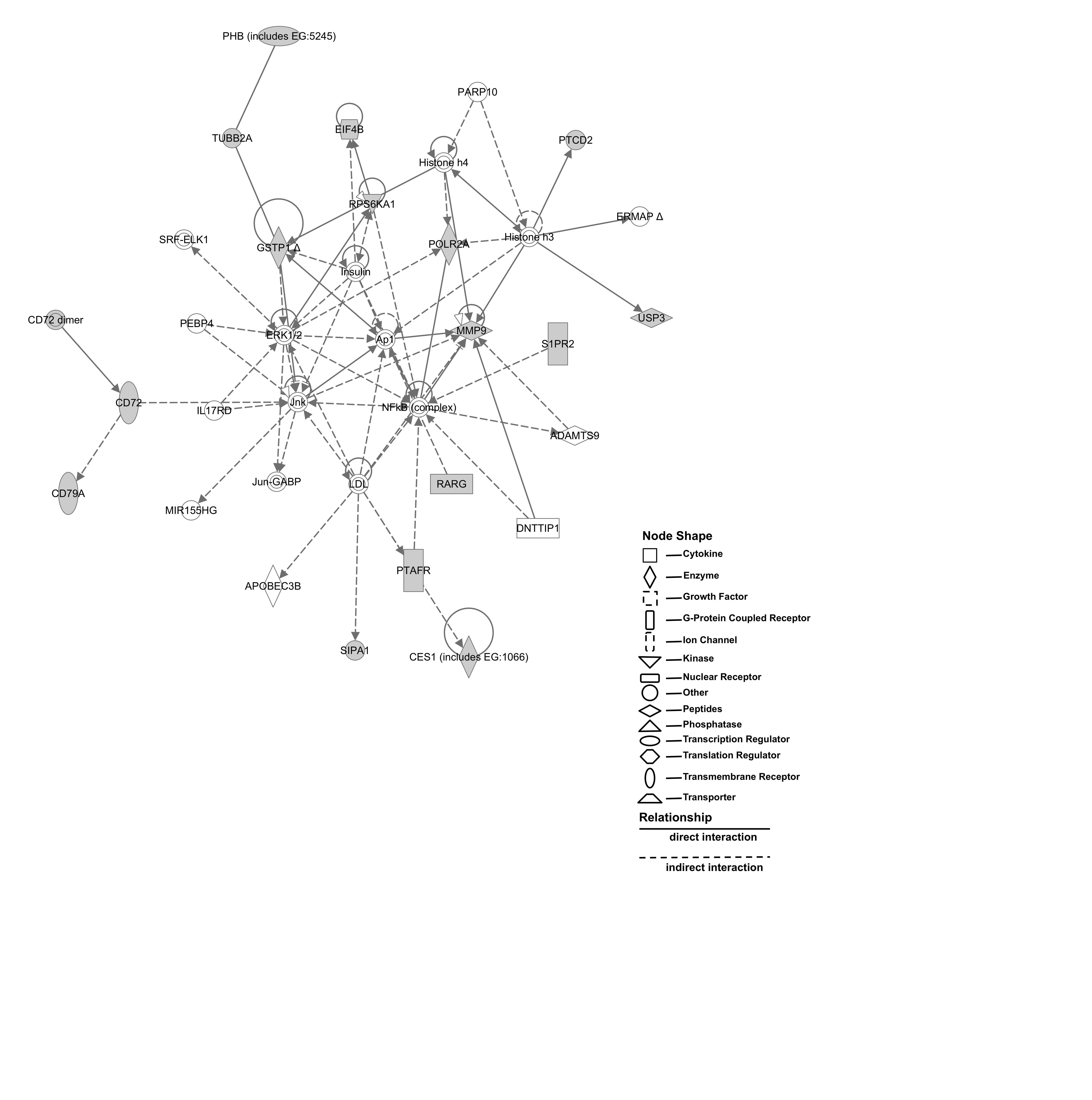

Supplement: Additional file 6 — Figure S2. Network built using differentially expressed genes in L. amazonensis-infected macrophages from C57BL/6 and CBA mice. C57BL/6 and CBA macrophages were cultured separately, then infected and processed for microarray analysis as described in Materials and Methods. The cell cycle network was modeled using IPA®. Genes marked in gray represent those found to be differentially expressed between C57BL/6 and CBA infected macrophages, while unmarked genes were added by IPA® due to a high probability of involvement in this network. Similar to Figure 2, the above network is displayed as a series of nodes (genes or gene products) and edges (or lines, corresponding to biological relationships between nodes). Nodes are displayed using shapes as indicated in the key. Solid lines denote direct interactions, whereas dotted lines represent indirect interactions between the genes represented in this network. [file 1471-2180-12-22-S6.TIFF]
